# *DNAH10* mutation correlates with cisplatin sensitivity and tumor mutation burden in small-cell lung cancer

**DOI:** 10.18632/aging.102683

**Published:** 2020-01-20

**Authors:** Man Li, Anqi Lin, Peng Luo, Weitao Shen, Dan Xiao, Lanying Gou, Jian Zhang, Linlang Guo

**Affiliations:** 1 Department of Pathology Zhujiang Hospital, Southern Medical University, Guangzhou 510282, People's Republic of China; 2 Department of Oncology, Zhujiang Hospital, Southern Medical University, Guangzhou 510282, People's Republic of China

**Keywords:** cisplatin, resistance, small cell lung cancer, *DNAH10*, tumor mutation burden

## Abstract

Chemotherapies based on platinum have been the standard first-line treatment for patients with small-cell lung cancer(SCLC) in the past years. However, the progression of patients occurs mostly due to rapid development of resistance to chemotherapy. In addition, the mechanisms involved in development of cisplatin-resistance in SCLC remain undetermined. Here, we analyzed whole-exome sequencing(WES) datasets from Genomics of Drug Sensitivity in Cancer(GDSC, N=55) and WES data and overall survival(OS) from a published cohort(N=101) to search for cisplatin-resistant target genes and genes associated with poor prognosis. We use our cohort(NCT03162705) as the validation set. We applied single sample gene set enrichment analysis(ssGSEA) to explore the potential molecular mechanisms of cisplatin-resistance. DNAH10 mutations in SCLC was significantly associated with cisplatin-resistance(P=0.0350), poor OS(HR:3.445;P=0.00035) and worse progression-free survival (PFS)(P=0.0142). ssGSEA showed that the negative regulation of FGFR, the SPRY regulation of FGF, and the positive regulation of noncanonical WNT and PI3K/AKT/IKK signaling pathways are differentially up- or downregulated in DNAH10-mutated cell lines. A higher TMB was observed in DNAH10-mutated cell lines. Taken together, DNAH10 mutations may have a potential value in prediction of cisplatin resistance and poor survival in SCLC. Moreover, DNAH10 mutations may have a positive correlation with high TMB in SCLC.

## INTRODUCTION

Small-cell lung cancer (SCLC) is a highly invasive neuroendocrine tumor characterized by rapid growth and early metastasis and accounts for about 15%–20% of lung cancers [1, 2]. Studies have confirmed that more than 90% of patients with SCLC have a history of smoking, and the occurrence of the disease is significantly associated with tobacco exposure [3]. In the past three decades, limited progress has been achieved in treatment of extensive-stage SCLC (ES-SCLC), and standard chemotherapy has adopted a two-drug combination with etoposide and cisplatin (four to six cycles). Although ES-SCLC usually achieves good results in the initial stage of platinum-based chemotherapy, the clinical response rate is 50%–75%, and the median overall survival (OS) is about 10 months [1, 4]. In addition, the majority of patients undergo relapse and develop chemoresistance within one year; some of them have primary resistance to cisplatin, and those with cisplatin sensitivity gradually develop resistance during the treatment [3, 5]. The development of drug resistance is due to the nonspecificity of cisplatin and the intracellular action and diversity of cisplatin-induced DNA-induced apoptosis. The resistance of tumor cells to cisplatin can be explained by various mechanisms [6–9], such as reduction of drug accumulation, enhancement of drug deactivation, promotion of DNA damage repair, inhibition of DNA damage response and changes in signaling pathways, and indirect regulatory factors of the apoptotic pathway. In addition, highly tumorigenic cancer stem cells (CSC) may lead to tumor recurrence and cisplatin resistance [10, 11]. Therefore, an in-depth understanding of cisplatin resistance mechanisms will provide new information for discovering potential therapeutic targets and improving the effectiveness of SCLC diagnosis.

Next-generation sequencing allows understanding of SCLC from the perspective of biologic drivers and molecular pathogenesis [1]. SCLC has no targetable driver oncogenes [3]. In SCLC, the mutations of genes, such as MYC proto-oncogene, may serve as molecular markers of rapid tumor progression and poor prognosis. In addition, no other gene mutations can be used as predictive and prognostic markers of platinum resistance and survival in SCLC [12].

Gene mutations are one of the molecular mechanisms of cisplatin resistance [12]. Sakai et al. [13] indicated that the compensatory mutations of BRCA1/2 genes can restore the homologous recombination (HR) ability of cells and make them more susceptible to cisplatin resistance. Human homologues of DNA mismatch repair (MMR) genes mutations, such as MLH1 and MSH2 mutations, are also associated with the development of acquired resistance to cisplatin [14, 15]. Lakin et al. [16] showed that p53 mutations may produce cisplatin resistance by disrupting the G1 phase of the cell cycle.

We analyzed the whole-exome sequencing (WES) datasets, the drug response data of Genomics of Drug Sensitivity in Cancer (GDSC) database, and the reported WES and corresponding clinical data of patients with SCLC (reported by George et al [17]) to screen gene mutations that are associated with primary resistance to cisplatin and poor prognosis. We also explored the potential mechanism for promoting cisplatin resistance in SCLC. Results showed that DNAH10 mutations may be a novel chemo-resistant gene that regulates primary cisplatin resistance and poor survival prognosis. Moreover, DNAH10 mutation may serve as molecular markers of TMB in SCLC. Hence, DNAH10 mutation can predict platinum drug sensitivity and survival prognosis and aid in developing optimal treatment modalities.

## RESULTS

### DNAH10 is mutated in cisplatin-resistant SCLC cell lines and correlates with prognosis

**Figure 1:**
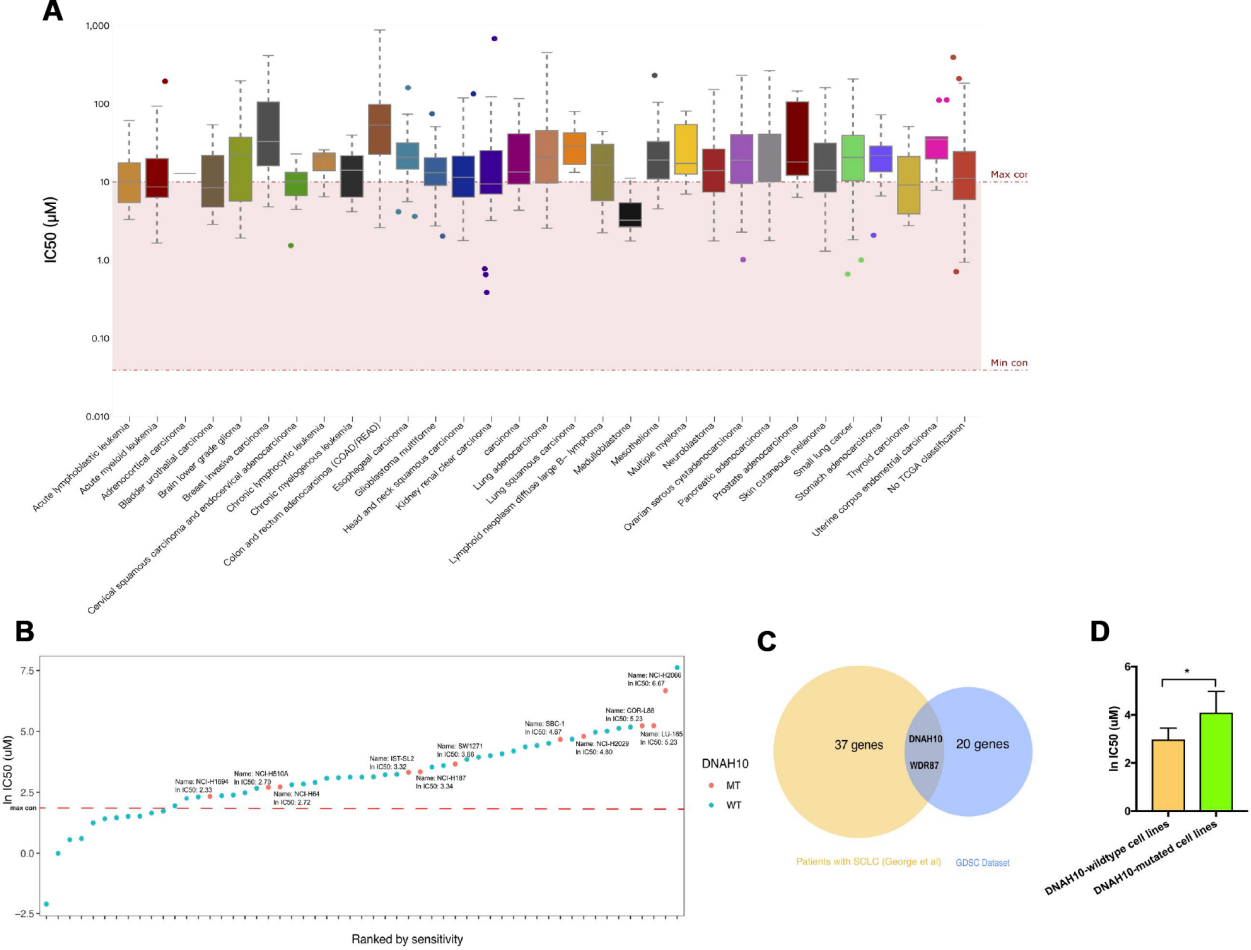
(**A**) IC50 distribution for cisplatin by tissue type. (**B**) Scatter plot of IC50 distribution for cisplatin in 55 SCLC cells. The red line shows the maximum screening concentration of 10.0 μM. DNAH10 mutant cell lines are highlighted in red. (**C**) Venn diagram showing the overlapping among genes predicted using the GDSC dataset and 101 patients with SCLC (reported by George et al). Abbreviation: IC50: half maximal inhibitory concentration; SCLC: Small-cell lung cancer; GDSC: The Genomics of Drug Sensitivity in Cancer Project (**D**) IC50 values for cisplatin in GDSC-SCLC cell lines with or without DNAH10 mutation. SCLC: Small-cell lung cancer; GDSC: The Genomics of Drug Sensitivity in Cancer Project.

**Figure 2:**
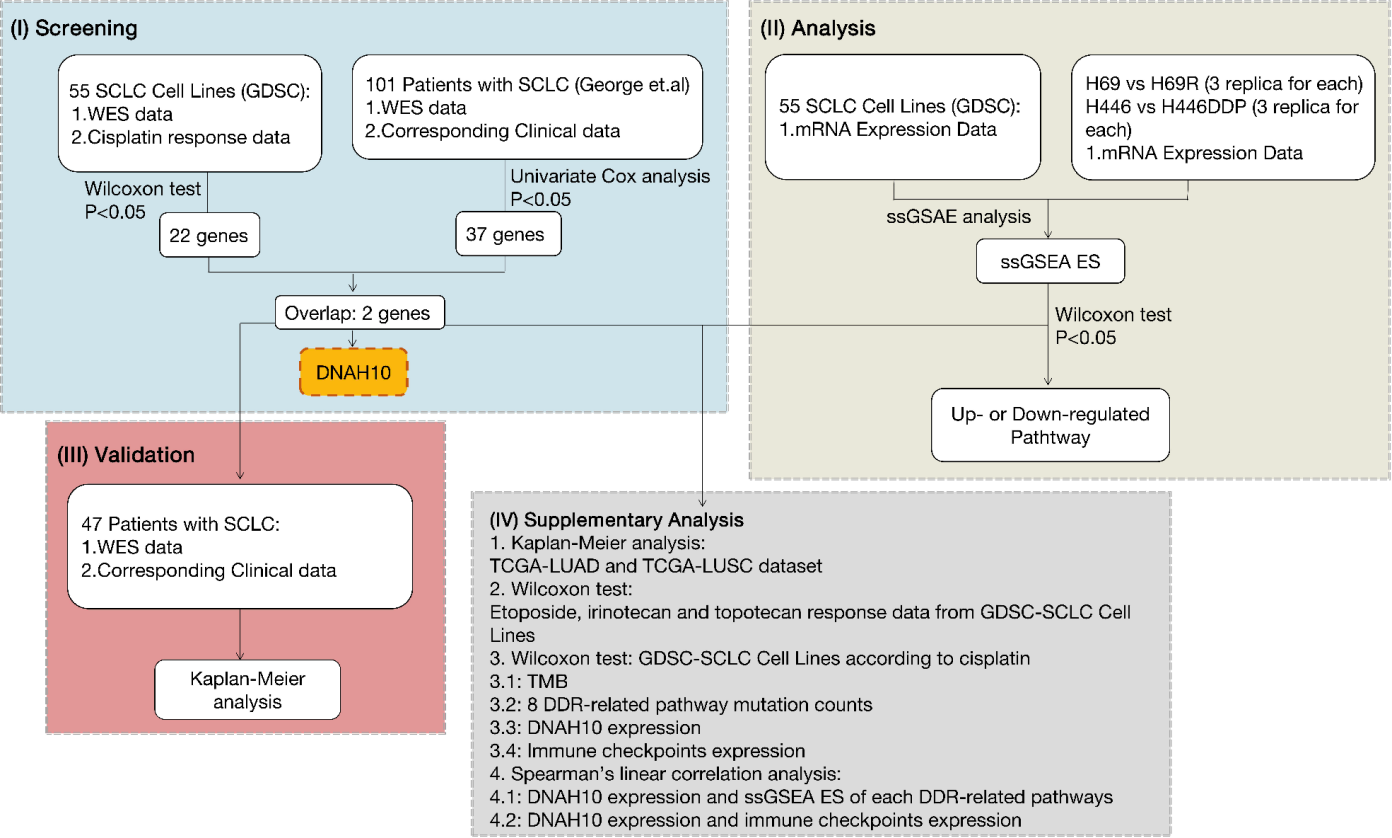
**Workflow of bioinformatics analysis.** SCLC: Small-cell lung cancer; GDSC: The Genomics of Drug Sensitivity in Cancer Project; TCGA: The Cancer Genome Atlas; WES: whole-exome sequencing; DDR: DNA damage response and repair; ssGSEA ES: ssGSEA: single sample gene-set enrichment analysis enrichment score; LUAD: Lung adenocarcinoma; LUSC: Lung squamous cell carcinoma; TMB: Tumor mutational burden.

**Figure 3:**
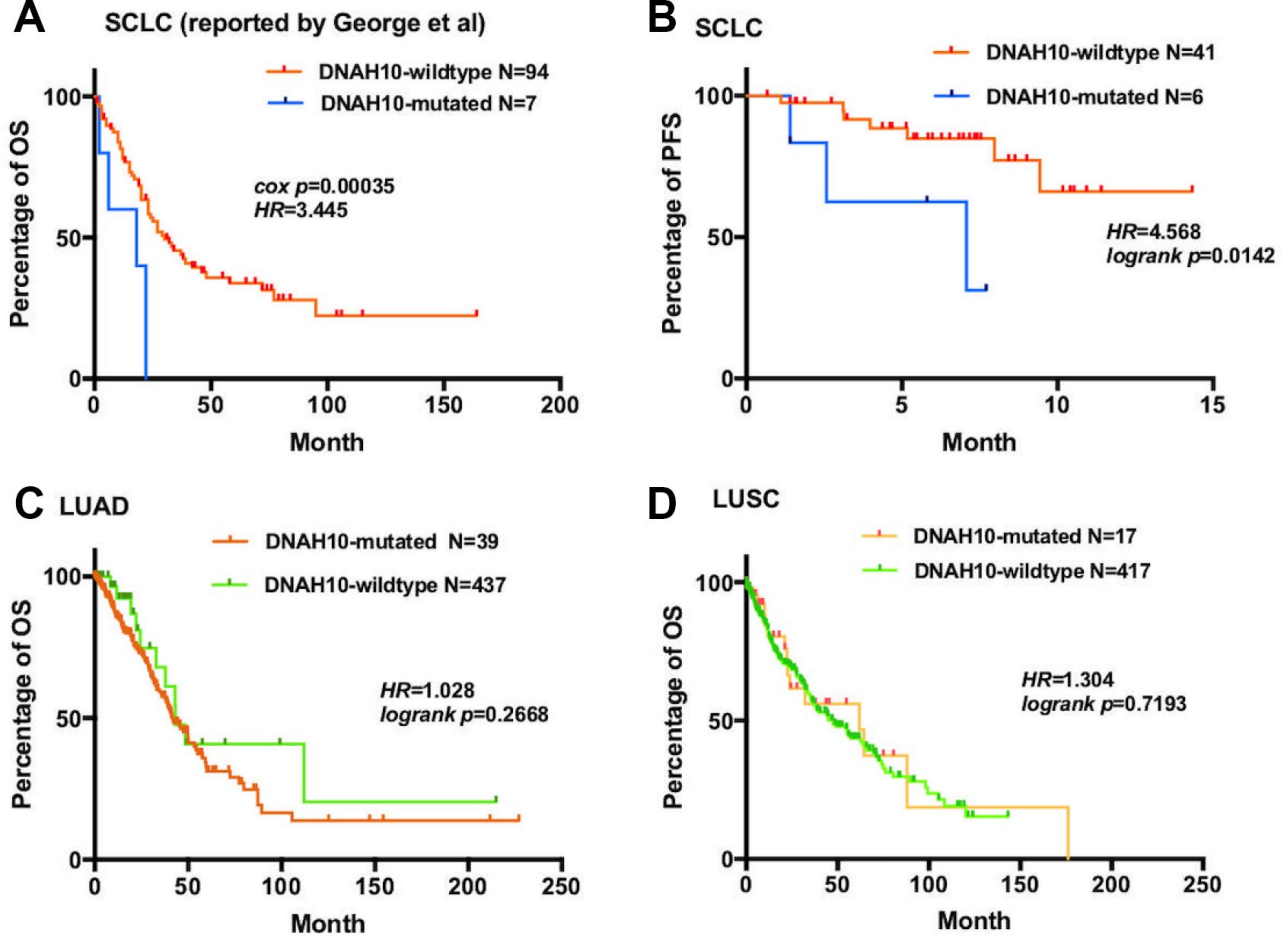
(**A**) For OS, Kaplan–Meier method revealed DNAH10 mutation (blue) and wild type (red) in the dataset of 101 patients with SCLC (reported by George et al); (**B**) For PFS, Kaplan–Meier method revealed DNAH10 mutations (blue) and wild type (red) in SCLC (NCT03162705). (**C**) For OS, Kaplan–Meier method showed DNAH10 mutations (red) and wild type (green) in the TCGA-LUAD dataset; (**D**) For OS, Kaplan–Meier method revealed DNAH10 mutations (red) and wild type (green) in the TCGA-LUSC dataset; SCLC: Small-cell lung cancer; TCGA: The Cancer Genome Atlas; WES: whole-exome sequencing; LUAD: Lung adenocarcinoma; LUSC: Lung squamous cell carcinoma; PFS: progression-free survival; OS: overall survival.

GDSC characterized about 1000 human cancer cell lines and screened them with 100s of compounds. Using this database, we obtained the IC50 distribution for cisplatin by tissue type (Figure 1A). We also selected 55 SCLC cell lines to identify the genomic markers of cisplatin sensitivity in SCLC. An ln IC50 ≥ 2.30 μM was regarded as cisplatin resistance, and an ln IC50 < 2.30 μM was regarded as cisplatin sensitivity in accordance with the standards of the GDSC. The characteristics of SCLC cell lines in GDSC databases are summarized in Supplementary Table 1. The majority of SCLC cell lines (76.36%, 42/55) were regarded as cisplatin-resistant, and 13 SCLC cell lines were regarded as cisplatin-sensitive (Figure 1B). Therefore, we studied the role of gene mutations in cisplatin resistance and survival prognosis in SCLC. Figure 2 shows the workflow of bioinformatics analysis. Twenty-two gene mutations, including DNAH10 and WDR87 mutations, were associated with cisplatin response in 55 GDSC-SCLC cell lines (P <0.05; Supplementary Table 2). We then assessed the impact of gene mutations on OS by using Cox regression of the published WES datasets (George et al. [17]) from 101 patients with SCLC. Thirty-seven gene mutations were found to be associated with OS (P <0.05; Supplementary Table 3). DNAH10 and WDR87 mutations (log-rank P =0.00035 and 0.0027, respectively) were detected in the published WES datasets (George et al). Finally, we identified a novel candidate gene, namely, DNAH10, from the GDSC database and the published datasets (Figure 1C). DNAH10-mutated cell lines showed significantly increased cisplatin resistance compared with the wild type (Figure 1D; P = 0.035). Kaplan–Meier survival analysis showed that SCLC with DNAH10 mutations had worse OS than that with wild-type DNAH10 (Figure 3A). We further found that DNAH10-mutated cell lines were cisplatin resistant (Figure 1B).

To further validate our hypothesis, we assessed DNAH10 mutation in our cohort comprising 47 patients with ES-SCLC who had not undergone any treatment. Patients with DNAH10 mutation had poor progression-free survival (PFS) (P = 0.0142, HR = 4.568; Figure 3B). Hence, DNAH10 mutation might be involved in chemosensitivity and prognosis of patients with SCLC. Moreover, no significant difference in OS was found in patients with The Cancer Genome Atlas Lung Adenocarcinoma (TCGA-LUAD) or The Cancer Genome Atlas Lung Squamous Cell Carcinoma (TCGA-LUSC) (P = 0.2668 vs. P = 0.7193, respectively, Figure 3C–3D) with and without DNAH10 mutation.

### Mutation profile or DNAH10 mutation status

**Figure 4:**
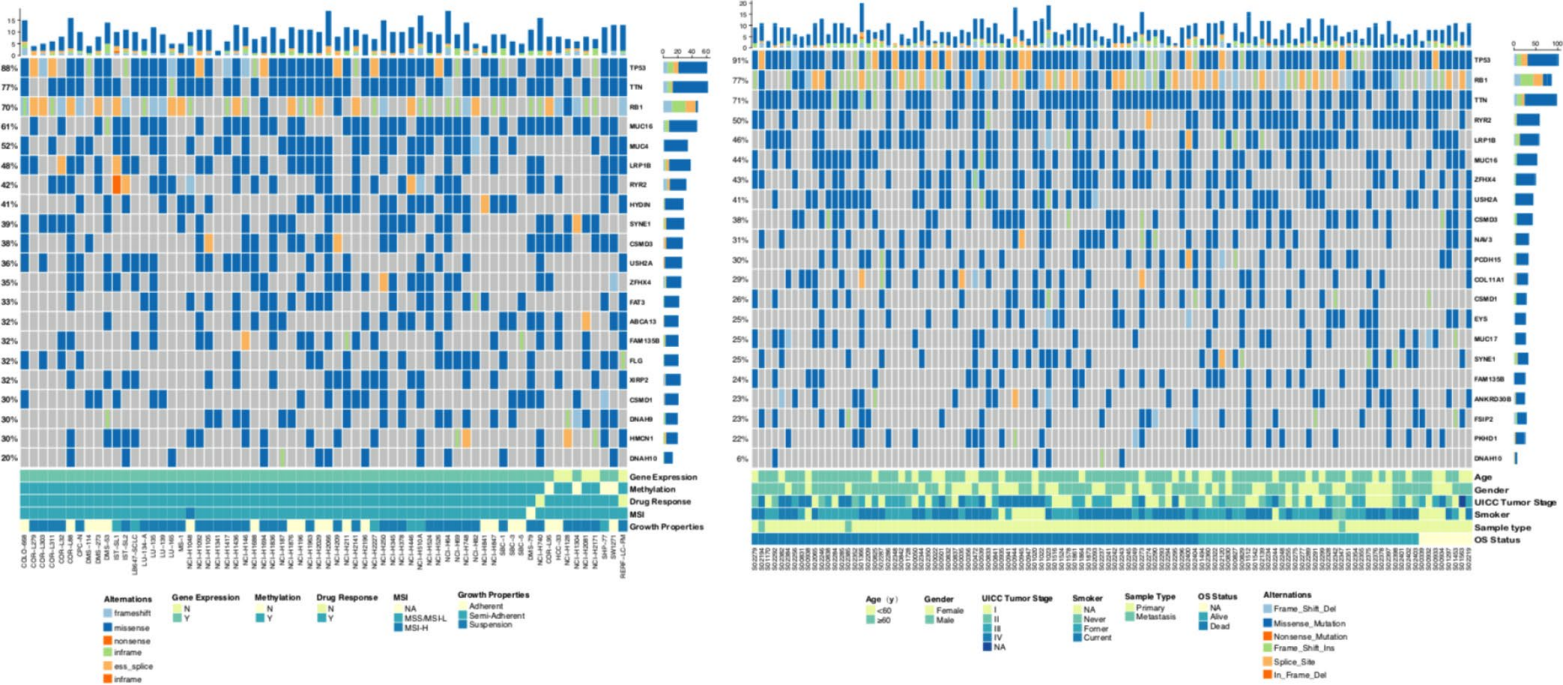
**Genomic alterations in SCLC.** (**A**) Sixty-six SCLC cell lines are arranged from left to right. Alterations in the SCLC cell line genes are annotated for each sample according to the color panel below the image. Details of 66 cell lines are displayed in the bottom panel. (**B**) Tumor samples from 101 patients with SCLC (reported by George et al.) are arranged from left to right. Alterations in the SCLC candidate genes are annotated for each sample according to the color panel below the image. Clinical information for each candidate gene is plotted on the bottom panel. SCLC: Small-cell lung cancer; MSI: Microsatellite instability; N: NO; Y: YES.

In the mutation landscape (Figure 4) of 66 SCLC cell lines and 110 patients with SCLC, we found the highest mutation frequencies for TP53, TTN, and RB1. TP53 and TTN mutations were mainly caused by missense, and RB1 mutation was mainly due to nonsense and splice [17]. Current studies have shown that SCLC has no clear target–driver gene mutations, and almost all SCLCs have none or inactivated TP53 and RB1 [17] Thirteen DNAH10 mutations were identified in 66 (19.7%) SCLC cell lines, and six mutations were found in 110 (5.50%) patients with SCLC. The mutation landscape for 56 SCLC cell lines (all have cisplatin-response data) is shown in Supplementary Figure 1. Detailed information of DNAH10 mutation identified in patients (reported by George et al [17].) and GDSC-SCLC cell lines is presented in Supplementary Table 4. The mutation plots of DNAH10 in patients (reported by George et al [17].) are shown in Supplementary Figure 2.

### Clinical characteristics of patients with SCLC

The characteristics of 101 patients with SCLC (George et al [17].) are listed in Table 1. No difference in the clinical expression was observed at enrollment between patients with and without DNAH10 mutation. From the 101 patients analyzed, 7 (6.93%) had mutated DNAH10 and 94 (93.7%) had wild-type DNAH10. The mean ages of diagnosis were 65.50 and 66.18 in patients with DNAH10 mutation and wild-type DNAH10, respectively (P = 0.383). In the study cohort, three (2.97%) patients had distant metastasis during follow-up: DNAH10-wildtype (n = 3). Smoking status was reported for 93.1% (n = 94) of the patients; 40.59% (n = 41) had smoking history amounting to a median of 43.78 pack/year. No statistically significant difference in pack/year (P = 0.816) and gender (P = 0.243) was found between patients with DNAH10 mutation and wild type. As predicted, the majority of the patients presented with advanced stages (III and IV) of the disease. The duration of OS was calculated from date of pathologic diagnosis until death or until the date of the last follow-up visit.

**Table 1:** Characteristics of small cell lung cancer patients (published by George et al).

**Characteristic**	**N=101**	**DNAH10 (+) N=7**	**DNAH10 (-) N=94**	**P value**
Age, years, mean±SD	66.16±8.15	65.50±7.78	66.18±8.21	0.383
Gender, N (%)				0.243
Male	64 (63.37%)	3 (42.86%)	61 (64.89%)	
Female	37 (36.63%)	4 (57.14%)	33 (35.11%)	
Missing	0 (0.00%)	0 (0.00%)	0 (0.00%)	
Smoking Status, N (%)				0.332
Never	2 (1.98%)	0 (0.00%)	2 (2.13%)	
Former	41 (40.59%)	1 (14.29%)	40 (42.55%)	
Current	51 (50.50%)	5 (71.43%)	46 (48.94%)	
Missing	7 (6.9%)	6 (5.94%)	1 (0.99%)	
Pack-years, mean±SD	43.78±22.53	41.00±1.41	43.86±22.86	0.861
Metastasis, N (%)				0.631
No	98 (97.03%)	7 (100.00%)	91 (96.81%)	
Yes	3 (2.97%)	0 (0.00%)	3 (3.19%)	
Missing	0 (0.00%)	0 (0.00%)	0 (0.00%)	
Stage, N (%)				0.551
I	34 (33.66%)	1 (14.29%)	33 (35.11%)	
II	21 (20.79%)	1 (14.29%)	20 (21.28%)	
III	27 (26.73%)	3 (42.86%)	24 (25.53%)	
IV	19 (18.81%)	2 (28.57%)	17 (18.09%)	
Missing	0 (0.00%)	0 (0.00%)	0 (0.00%)	

### Identification of differentially expressed genes (DEGs) among GDSC-SCLC cell lines

**Figure 5:**
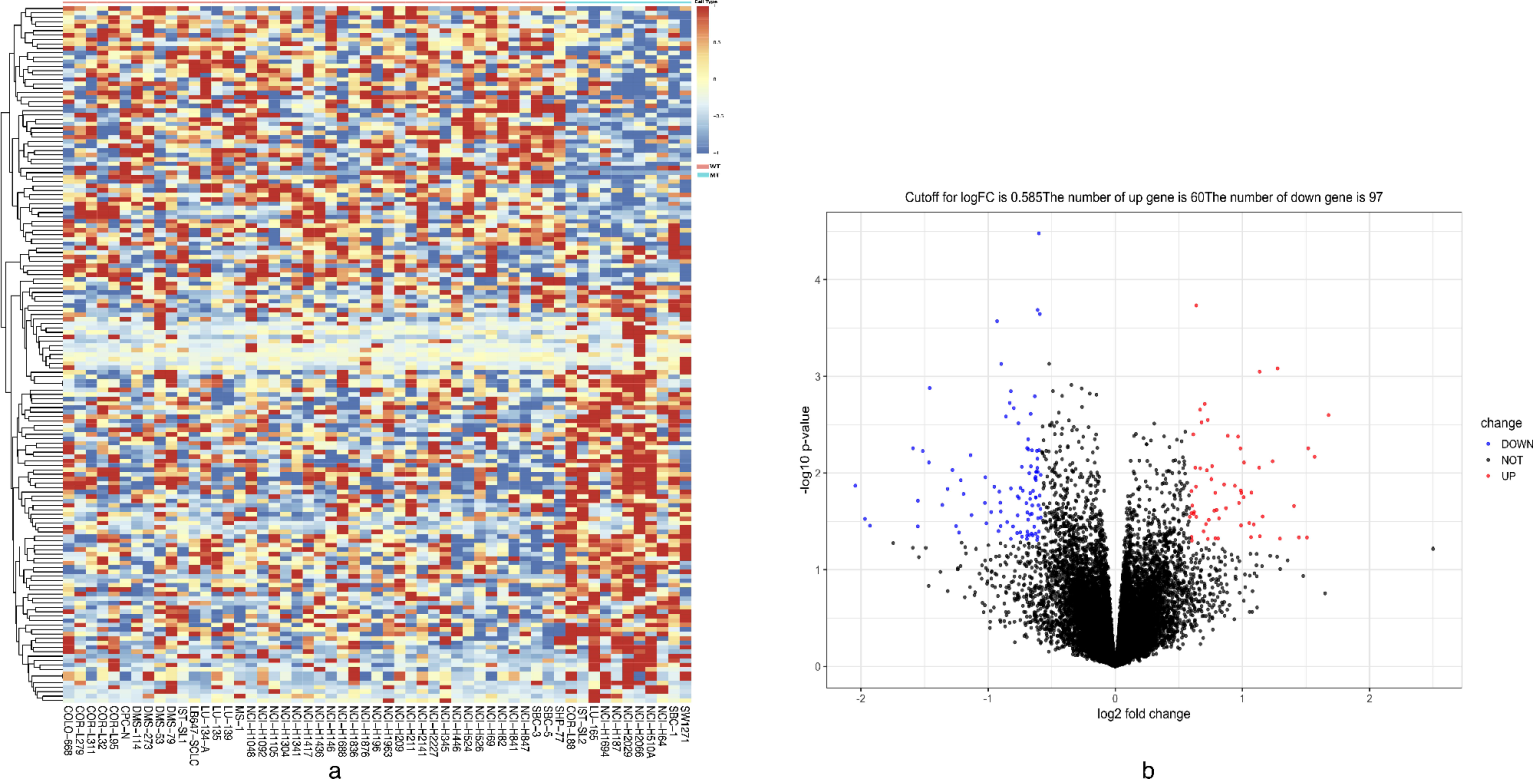
**Heatmap and volcano plot showing DEGs.** (**A**) Heatmap of the all 157 DEGs between DNAH10-mutated and wild-type DNAH10 SCLC cell lines [60 upregulated genes (red) and 97 downregulated genes (blue)]. (**B**) Volcano plot of differentially expressed mRNAs between DNAH10-mutated and wild-type DNAH10 SCLC cell lines. Red indicates high expression, and green indicates low expression (|FC| >1.5 and P <0.05). DEGs were calculated using the limma package in R software (Version.3.6). Sixty highly expressed DEGs and 97 low expressed ones. The volcano plot was conducted by the ggplot2 package of R language. DEGs: differentially expressed genes; FC: fold change; SCLC: Small Cell Lung Cancer.

The mRNA expression matrix files of GDSC-SCLC cell lines were analyzed in R software by the limma package. The P-values < 0.05 and FC was ≥3/2 or ≤ 2/3. were chosen as cut-off criteria. A heatmap of all 157 DEGs is shown in Figure 5A. Compared with cisplatin-sensitive GDSC-SCLC cell lines, 60 upregulated and 97 downregulated DEGs were observed in cisplatin-resistant GDSC-SCLC cell lines (Figure 5B, Supplementary Table 5).

### ssGSEA identifies DNAH10-related signaling pathways

**Figure 6:**
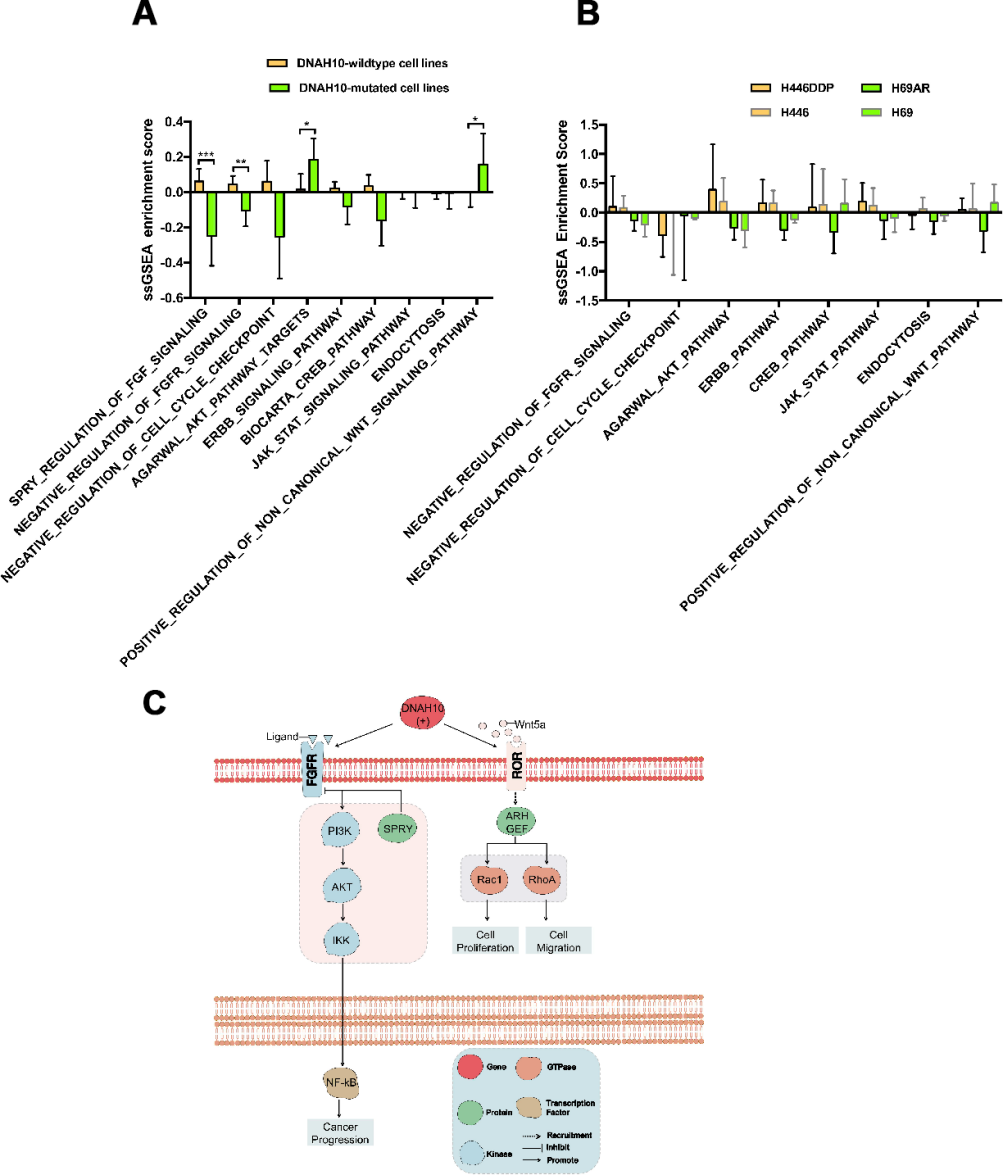
(**A**) ssGSEA enrichment scores (ES) of 55 SCLC cell lines in GDSC. (**B**) Potential mechanism of DNAH10 mutation to predict the resistance of cisplatin in SCLC. (**C**) ssGSEA ES of 12 human SCLC cell lines in three replicates. No significant difference was found between each group. ssGSEA: single sample gene-set enrichment analysis; SCLC: Small Cell Lung Cancer. ***P <0.001; **P <0.01; *P <0.05.

To understand potential DNAH10-related pathways, we conducted ssGSEA among 55 GDSC-SCLC cell lines (Supplementary Table 6) followed by Mann–Whitney U test with ssGSEA ES (Supplementary Table 7). We found significant difference in the contents of pathways, such as negative regulation of FGFR (fibroblast growth factor receptor) (P = 0.0060), SPRY (sprouty RTK signaling antagonist) regulation of FGF (fibroblast growth factor) (P < 0.0001), and positive regulation of noncanonical WNT (P = 0.0411) and PI3K/AKT/IKK signaling pathway (P = 0.0433) between DNAH10 mutation and wild-type (Figure 6A). We then selected H69/H69AR and H446/H446DDR to assess ssGSEA ES. The significantly DNAH10-related pathways among GDSC-SCLC cell lines were not found between H69/H69AR and H446/H446DDR (Figure 6B).

### Tumor mutation burden is significantly associated with DNAH10 mutation status

**Figure 7:**
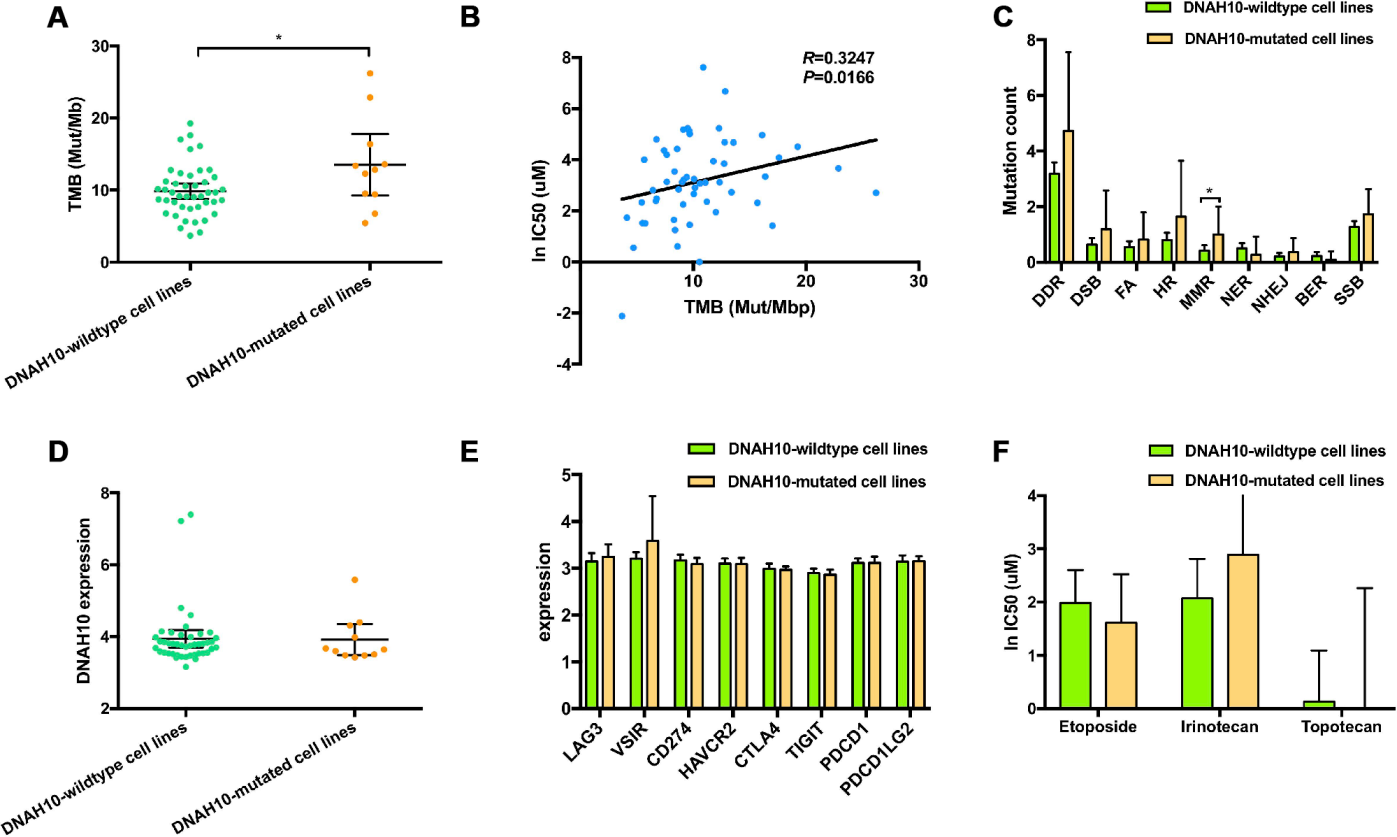
(**A**) Distribution of TMB based on the DNAH10 mutation status of 55 SCLC cell lines in the GDSC database. (**B**) Correlation between TMB and WES and ln IC50 values of 55 SCLC cell lines in GDSC (P = 0.0166; Sprearman r = 0.3247). (**C**) Trend toward increased mutational counts in DDR-related pathway in patients with DNAH10 mutation. (**D**) Distribution of DNAH10 expression based on DNAH10 mutation status of 55 SCLC cell lines in the GDSC database. (**E**) Trend toward increased immune checkpoint expression in patients with DNAH10 mutation. SCLC: Small-cell lung cancer; GDSC: The Genomics of Drug Sensitivity in Cancer Project; (**F**) ln IC50 values of three drugs based on DNAH10 mutation status of SCLC cell lines in GDSC. SCLC: Small-cell lung cancer; GDSC: The Genomics of Drug Sensitivity in Cancer Project; FA, Fanconi anemia; HR, homologous recombination; NHEJ, non-homologous end joining; BER, base excision repair; MMR, mismatch repair; NER, nucleotide excision repair; DSB, double strand breaks; SSB, single strand breaks; TMB, tumor mutational burden.

TMB was higher in patients with DNAH10 mutation compared with patients without the mutation (median TMB: 12.84 vs. 9.41 Mut/Mb, Mann–Whitney P = 0.0476, Figure 7A). A strong correlation was found between increased TMB and increased IC50 values of cisplatin (Spearman ρ=0.3247, P = 0.0166; Figure 7B).

### Additional features associated with DNAH10 mutation status

Analyses of several tumor types, including urothelial carcinoma, triple negative breast cancer, and ovarian cancer, have reported associations between altered genes in DNA damage response and repair (DDR)-related pathways and response or resistance to platinum compound. DNAH10-mutated cell lines had significantly higher mutation count in the mismatch repair (MMR) pathway compared with the wild-type DNAH10 cell lines (Mann–Whitney P < 0.05; Figure 7C). Moreover, the DNAH10-mutated cell lines showed higher genomic alternations in the DDR pathway than the wild-type cells lines, but the difference was not statistically significant (Figure 7C). No significant difference in the distribution of DNAH10 expression and common immune checkpoints expression values was found in GDSC-SCLC cell lines with or without DNAH10 mutation (Figure 7D and 7E, respectively). Furthermore, there was no statistically significant in the three other drugs' response between DNAH10 mutant and DNAH10 wildtype cell lines (Figure 7F; Supplementary Table 8).

## DISCUSSION

SCLC is sensitive to radiotherapy and chemotherapy, and first-line chemotherapy is highly effective for this disease3. The current standard first-line treatment for most patients with SCLC is a combination of etoposide and cisplatin (EP) or irinotecan and cisplatin (IP) [3]. A meta-analysis [18] showed that patients with SCLC who received the standard first-line treatment had an objective response rate (ORR) of 67%, PFS of 5.5 months, and OS of 9.6 months; however, SCLC relapsed and became resistant to the drug [19]. Moreover, cisplatin-resistant tumors developed tolerance to drugs that are completely unrelated to the mechanism of action of cisplatin [28]. The GDSC database showed that most SCLC cell lines (76.36%, 42/55) were resistant to cisplatin. Therefore, molecular markers for predicting cisplatin resistance can greatly improve treatment outcomes for SCLC.

In this study, we analyzed the WES and transcriptome data of 55 GDSC-SCLC cell lines as well as the cisplatin response, WES, and clinical data of 101 patients (reported by George et al [17]). We found that DNAH10 mutation may be associated with cisplatin resistance and poor survival of patients with SCLC. Based on the score of ssGSEA, DNAH10-mutated cell lines were negatively associated with the SPRY regulation of FGF signaling and the negative regulation of FGFR signaling (Figure 6C; P < 0.0001 and P = 0.0411, respectively). In addition, the score of PI3K/AKT/IKK signaling in DNAH10-mutated cell lines was significantly higher than that in the wild-type DNAH10 cell lines (P = 0.0433). The binding of FGF2 to FGFR can activate PI3K/AKT signaling and affect the cytotoxicity of chemotherapeutic drugs, ultimately influencing the sensitivity of tumor cells to chemotherapeutic drugs [20]. Song et al. [21] showed that FGF1/FGF2 overactivated FGFR signaling and promoted chemoresistance in NSCLC. In addition, FGF inhibitors increased the sensitivity of human prostate PC3 tumors to doxorubicin [22]. The SPRY (Sprouty) protein is an important inhibitor of receptor tyrosine kinase (RTK)-dependent signaling pathways and is found in several tumor types, such as prostate cancer, lung cancer, colon cancer, lymphoma, and thyroid cancer. The expression of each subtype of the SPRY protein either decreases or is deleted, and signaling pathways, such as RTK, is over-activated, resulting in abnormal proliferation of tumor cells and promoting the occurrence and development of tumors [23–27]. Agarwal et al. [28] showed that PI3K/AKT/IkB kinase (IKK) signaling was abnormally activated in colorectal cancer and upregulated the expression of MDR1 genes and its protein product P-glycoprotein (P-gp) by regulating the transcription factor NF-kB, eventually leading to chemoresistance. Ohta et al. [29] found that PI3K inhibitors enhanced the sensitivity of ovarian cancer to cisplatin by inhibiting p-AKT levels and downstream targets of PI3K/Akt cascades, (such as BAD and NF-κB). In addition, high expression of AKT1 or AKT2 and AKT3 indicated cisplatin resistance by modulating the threshold of several apoptotic pathways, such as increasing the expression of Bcl-xL (antiapoptotic Bcl-2 family member protein) and delaying the activation of the p53 pathway [30]. Furthermore, we found that the positive regulation of noncanonical Wnt signaling was highly activated in DNAH10-mutated cell lines (P = 0.0411). Yu et al. [31] found that in chronic lymphocytic leukemia, Wnt5a, a classical gene for noncanonical Wnt signaling, recruited guanine exchange factors by binding to tyrosine kinase-like orphan receptors (ROR1 and ROR2); the gene promoted tumor cell proliferation and metastasis by activating Rac1 and RhoA. Therefore, we hypothesized that the negative regulation of FGFR, the SPRY regulation of FGF, and the positive regulation of noncanonical WNT and PI3K/AKT/IKK signaling pathways may be the key pathway regulated by DNAH10 mutation for promoting cisplatin resistance in DNAH10-mutated SCLC cell lines. We further assessed the relationship between DNAH10 mutation and other chemotherapy drugs, but we did not find that DNAH10-mutated cell lines were more chemoresistant to three other chemotherapy drugs (Figure 7F). Moreover, no significant difference in OS was found between patients with TCGA-LUAD or TCGA-LUSC (P = 0.2668 and P = 0.7193. respectively, Figure 3C–3D) with and without DNAH10 mutation.

Platinum drugs form a complex with DNA, leading to DNA damage, which is recognized by the MMR system and leads to apoptosis [12]. Hypothetically, DDR pathway-damaged tumors could be more sensitive to platinum compounds [12]. The DNAH10 mutant had higher number of mutations in the MMR signaling than the wild-type DNAH10 GDSC-SCLC cell lines (Figure 7C; P <0.05) and was found in most DDR pathways. A trend for a higher number of mutations was also detected. Most tumors with genetic mutations in the DDR pathway had higher TMB due to accumulation of incorrect DNA damage [32]. A study found that TMB and MMR can be used as a biomarker for the prognosis of immune checkpoint inhibitors [33–35]. Check-Mate032 showed that the combination of nivolumab and nivolumab + ipilimumab was effective for SCLC with high TMB. This study speculated that TMB plays a molecular role in the efficacy of ICIs in SCLC [36]. The above studies suggest whether we can use DNAH10 mutations as molecular markers for predicting TMB in SCLC. In addition, we found that DNAH10 expression was positively correlated with the expression of immune checkpoints, such as LAG3, CTAL4, TIGIT, and HAVCR2 (Supplementary Figure 3A). However, we failed to find a correlation between DNAH10 expression and ssGSEA ES of each DDR pathway (Supplementary Figure 3B). We also analyzed two groups of human SCLC cell lines (H69 vs. H69AR; H446 vs. H446 DDP) to explore similarities between the primary and secondary resistance mechanisms of cisplatin by visualizing DEGs (Supplementary Figure 4). The ssGSEA ES of the 12 cell lines showed no significant difference in the possible signaling pathways in primary resistance of cisplatin (Figure 6B). Hence, DNAH10 may be involved in the regulation of primary resistance to cisplatin in SCLC.

This study presents some limitations. First, the precise molecular mechanisms of DNAH10 mutation on chemoresistance in SCLC remain unclear. Second, we did not compare the conservation of different mutation across evolution mainly because we found that the DNAH10 mutation sites are randomly occurred in different samples. Third, no animal models are available for providing further evidence for our hypothesis. In this regard, relevant animal models should be established to verify our hypothesis.

In conclusion, DNAH10 mutation might be a potential biomarker for cisplatin resistance and poor survival in patients with SCLC. Our results showed the good association of DNAH10 mutations with high TMB in SCLC. Moreover, the negative regulation of FGFR, the SPRY regulation of FGF, and the positive regulation of noncanonical WNT and PI3K/AKT/IKK signaling pathways may be the key pathways regulated by DNAH10 mutation for promoting cisplatin resistance in SCLC. If new lung cancer patients are diagnosed with SCLC, patients could be sequenced using WES or target sequencing (including DNAH10). With DNAH10 mutations were detected with next-generation sequencing assays or target sequencing, and its presence in the current analysis was strongly associated with cisplatin-resistance. clinicians can try not to use cisplatin, consider other combinations of chemotherapy; or consider using ICIs for first-line treatment of DNAH10 mutant SCLC. Our findings provide potential therapeutic targets for improving the efficacy of chemotherapy in patients with SCLC and DNAH10 mutation. Further experimental validation should be performed to prove the biological effect of DNAH10 mutation on SCLC.

## MATERIALS AND METHODS

### WES sequencing, gene expression, cisplatin response, and clinical data

We analyzed WES data (Illumina HiSeq 2000) of SCLC cell lines from Genomics of Drug Sensitivity in Cancer (GDSC, release-8.0, updated on July 2019) databases [37]. Fifty-five SCLC cell lines with at least two types of data including gene expression and cisplatin response were investigated. The WES data of patients with SCLC were obtained from the supplementary file of the study reported by George et al. Gene expression data (55 samples, Affymetrix Human Genome U219 Array, Robust Multichip Average) were downloaded from GDSC and analyzed. Clinical records of SCLC patients were also obtained from the supplementary file of the study reported by George et al.

### SCLC cell line collection, cDNA microarray, and mRNA sequencing

Human SCLC cell lines, namely, NCI-H69, NCI-H69AR, and NCI-H446, were acquired from the American Type Culture Collection (ATCC, USA). Chemo-resistant H446DDP cells were obtained by incubating H446 cells in progressively increasing cisplatin doses of up to 5 μg/mL for 6 months. RNA was extracted from human cell lines such as NCI-H69, NCI-H69AR, NCI-H446, and NCI-H446DDP by using Trizol method and quantified by Qubit3.0. RNA quality was assessed by 4200 TapeStation (Agilent Technologies). RNA libraries were prepared with VAHTSTMmRNA-seq V3 preparation kit (Illumine). The libraries were then quantified and sequenced with Illumina Novaseq6000 platform.

### Tissue sample collection

Forty-seven fresh–frozen SCLC samples were collected from patients between May 2017 and January 2019 (NCT03162705, details of protocol available at https://www.clinicaltrials.gov/). The patients received care and follow-up in Zhujiang Hospital (Southern Medical University, Guangzhou, China), the First Affiliated Hospital of Guangzhou Medical University (Guangzhou, China), the Collaborative Innovation Center for Cancer Medicine (Guangzhou, China), or the Nangfang Hospital (Southern Medical University, Guangzhou, China). Informed consent was obtained from all patients before specimen collection. The experiments were approved by the Ethics Committee of the Southern Medical University (Guangzhou, China).

### Library preparation and sequencing

Whole-genome sequencing was performed on 47 SCLC fresh–frozen tumor samples. For WES, genomic DNA from fresh–frozen tumor samples and whole blood control samples were extracted with QIAamp DNA FFPE Tissue Kit and DNeasy Blood and Tissue Kit (Qiagen), respectively. DNAs were quantified by Qubit 3.0 by using the dsDNA HS Assay Kit (ThermoFisher Scientific). Library preparations were performed with KAPA Hyper Prep Kit (KAPA Biosystems). The average coverage depth for tumors was set as 157×. Trimmomatic was used for FASTQ file quality control. Paired-end reads were aligned to the reference human genome (build hg19) by using Burrows–Wheeler Aligner. PCR deduplication was performed using Picard. Local realignment around indels and base quality score recalibration were performed using GATK3.

### Detection of biomarker genes

Predictive genes for patients’ survival and sensitivity to cisplatin were detected by three steps. (1) The association of gene mutations (mutant frequency>10% in GDSC databases) with the IC50 values of cisplatin was assessed by Wilcoxon test (P <0.05). (2) The association of gene mutations (mutant frequency>10%) with OS of patients with SCLC in a reported cohort was determined by univariate Cox proportional hazard regression model (P <0.05). (3) Common genes in the first and second steps were identified. The overall process yielded two predictive genes, namely, DNAH10 and WDR87, for SCLC tumors.

### Differential gene expression, functional enrichment analysis based on single sample gene set enrichment analysis (ssGSEA) scores

To investigate the genomic profile between DNAH10 mutation and wild-type groups, we performed differential expression gene (DEG) analysis in SCLC cell lines by the limma [38] package (GDSC databases; H69 vs. H69AR; H446 vs. H446DDP). P values were set as 0.05 and Fold change (FC) was ≥3/2 or ≤ 2/3. Volcano plots were drawn to visualize DEGs. ssGSEA is an extension of gene set enrichment analysis (GSEA), which calculates separate enrichment scores for each pairing of a sample and gene set [39]. ssGSEA was performed using GSVA [40] package from Bioconductor in R software (version.3.6) with C2 (curated gene sets) and C5 (GO gene sets) collections [41]. Each ssGSEA enrichment score (ES), which represents the degree to which genes in a particular gene set are coordinately up- or down-regulated within a sample, was presented in the output file.

### Statistical methods

For clinical data, ssGSEA ES, DNAH10 expression, immune checkpoint expression, IC50, TMB, and DNA damage response and repair (DDR)-related pathway alternation were compared between the two groups. Wilcoxon test/Mann–Whitney U test was used for data with abnormal distribution, and independent-sample Student’s t-test was used for data with normal distribution. Survival curves were estimated with R software (version.3.6) by using survival [42] and survminer [43] packages from Bioconductor. Differences in survival were assessed by log-rank test. R software, SPSS (IBM.version.23.0), and GraphPad Prism (version.7.0) were used for statistical analysis. Limma package19 was used to analyze the normalized gene expression data for the 55 SCLC cell lines (DNAH10 mutation vs. wild-type DNAH10) from the GDSC dataset. DEGseq2 [44] in R/Bioconductor software was applied to identify DEGs from the RNA-Seq data of 12 human SCLC cell lines (H69 vs. H69AR; H446 vs. H446DDP). FC ≥ 3/2 or FC ≤ 2/3 and P-values < 0.05 were considered statistically significant for the DEGs. Expression of the gene signature was visualized by heatmap and volcano plot. R package “ComplexHeatmap” [45] was employed to visualize mutation landscape of GDSC-SCLC cell lines and patients with SCLC (reported by George et al). Spearman correlation analysis was used to identify correlation between DNAH10 expression and ssGSEA ES. P-values < 0.05 were considered to indicate a statistically significant difference.

## Supplementary Material

Supplementary Figures

Supplementary Tables

Supplementary Table 1

Supplementary Table 2

Supplementary Table 3

Supplementary Table 4

Supplementary Table 5

Supplementary Table 6

Supplementary Table 7

Supplementary Table 8
