# Supplementary material for: DNAH10 mutation correlates with cisplatin sensitivity and tumor mutation burden in small-cell lung cancer
Source: Aging (Albany NY). 2020 Jan 20;12(2):1285–303. doi: 10.18632/aging.102683 (PMC7053592; doi:10.18632/aging.102683)
Supplement: Supplementary Figures [file aging-12-102683-s008..pdf]

SUPPLEMENTARY FIGURES

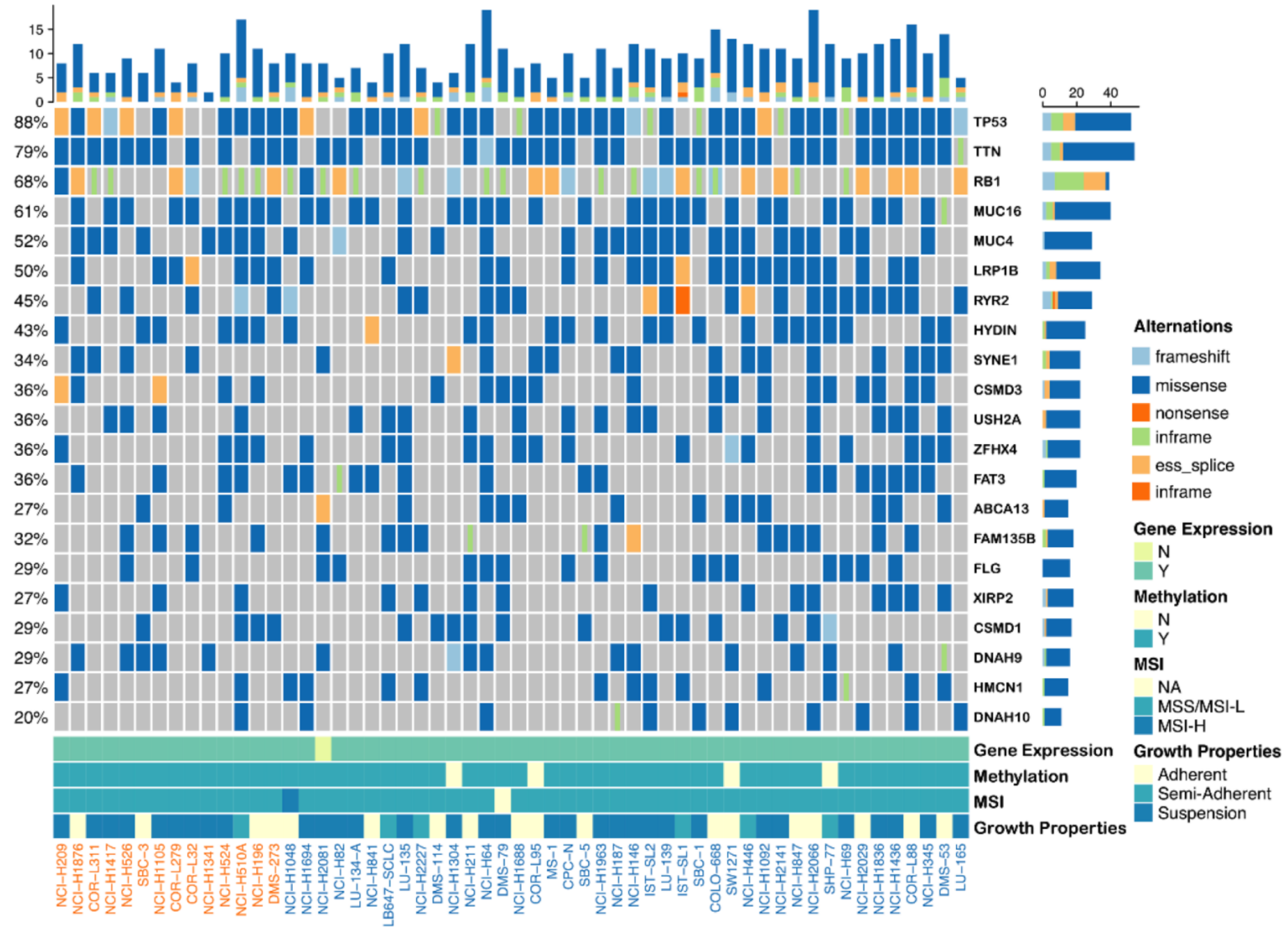

Supplementary Figure 1. Genomic alterations in 56 SCLC cell lines arranged according to their sensitivity to cisplatin (from the most sensitive to resistant). Alterations in the SCLC cell line genes are annotated for each sample according to the color panel below the image. Yellow represented cisplatin-sensitive SCLC cell lines, while blue represented cisplatin-resistant SCLC cell lines. Details of 56 cell lines are displayed in the bottom panel. SCLC: Small-cell lung cancer; MSI: Microsatellite instability; N: NO; Y: YES.

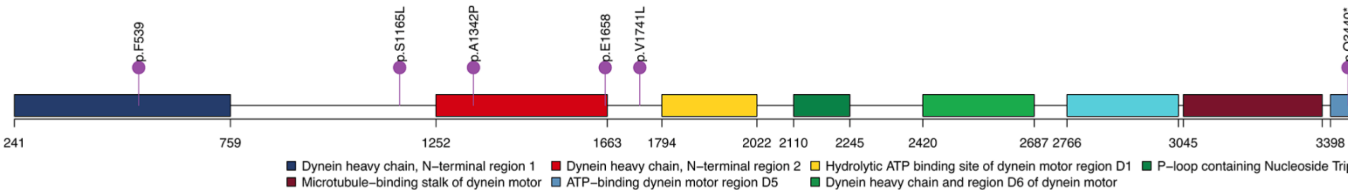

Supplementary Figure 2. Lollipop plot shows the distribution of DNAH10 mutations in patients (reported by George et al.).

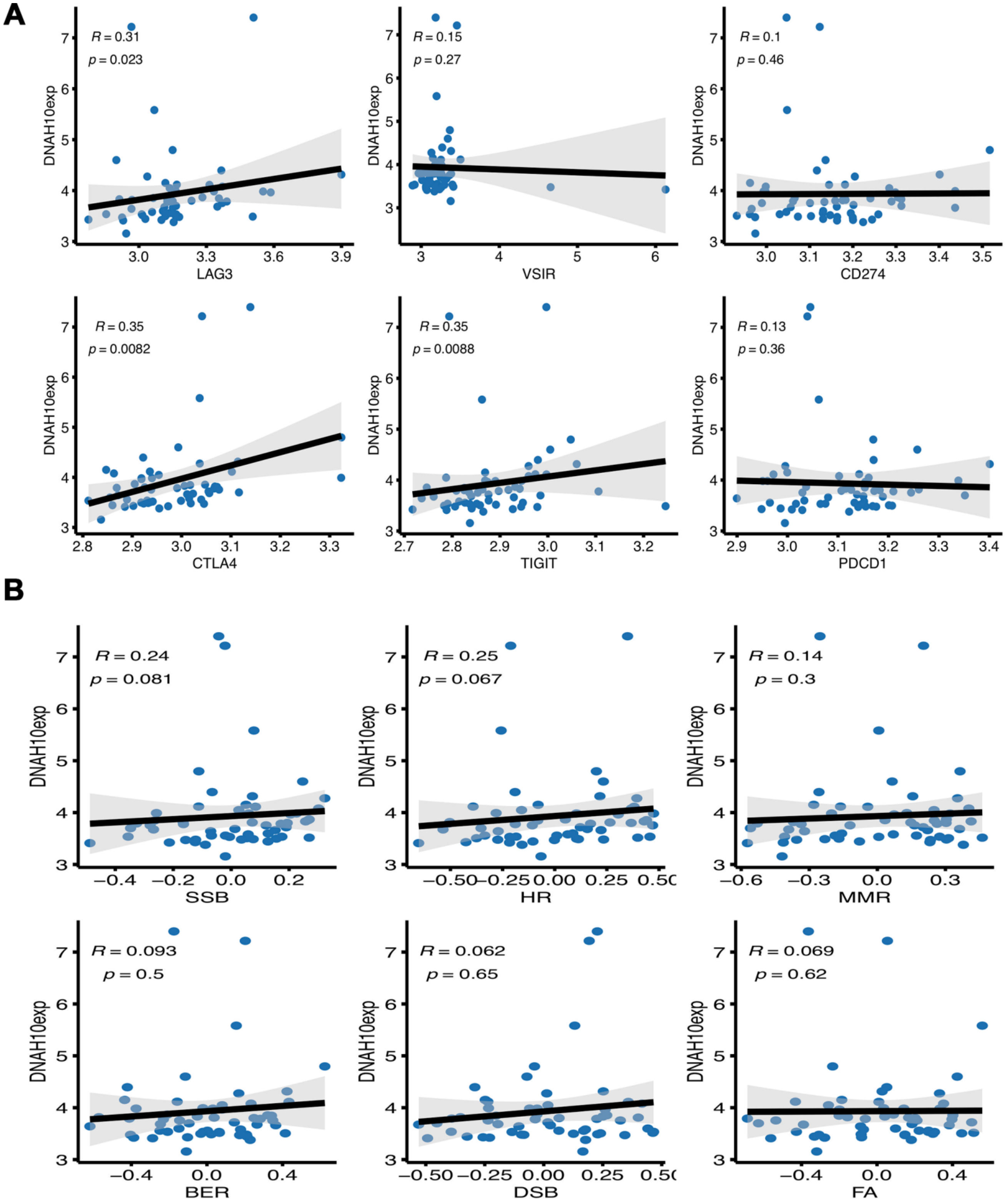

Supplementary Figure 3. (**A**) Correlation between DNAH10 expression and common immune checkpoints expression of 55 SCLC cell lines in the GDSC database. (**B**) Correlation between DNAH10 expression and ssGSEA ES of eight DDR-related pathways of 55 SCLC cell lines in the GDSC database. SCLC: Small-cell lung cancer; GDSC: The Genomics of Drug Sensitivity in Cancer Project; DDR: DNA damage response and repair; ssGSEA ES: ssGSEA: single sample gene-set enrichment analysis enrichment score

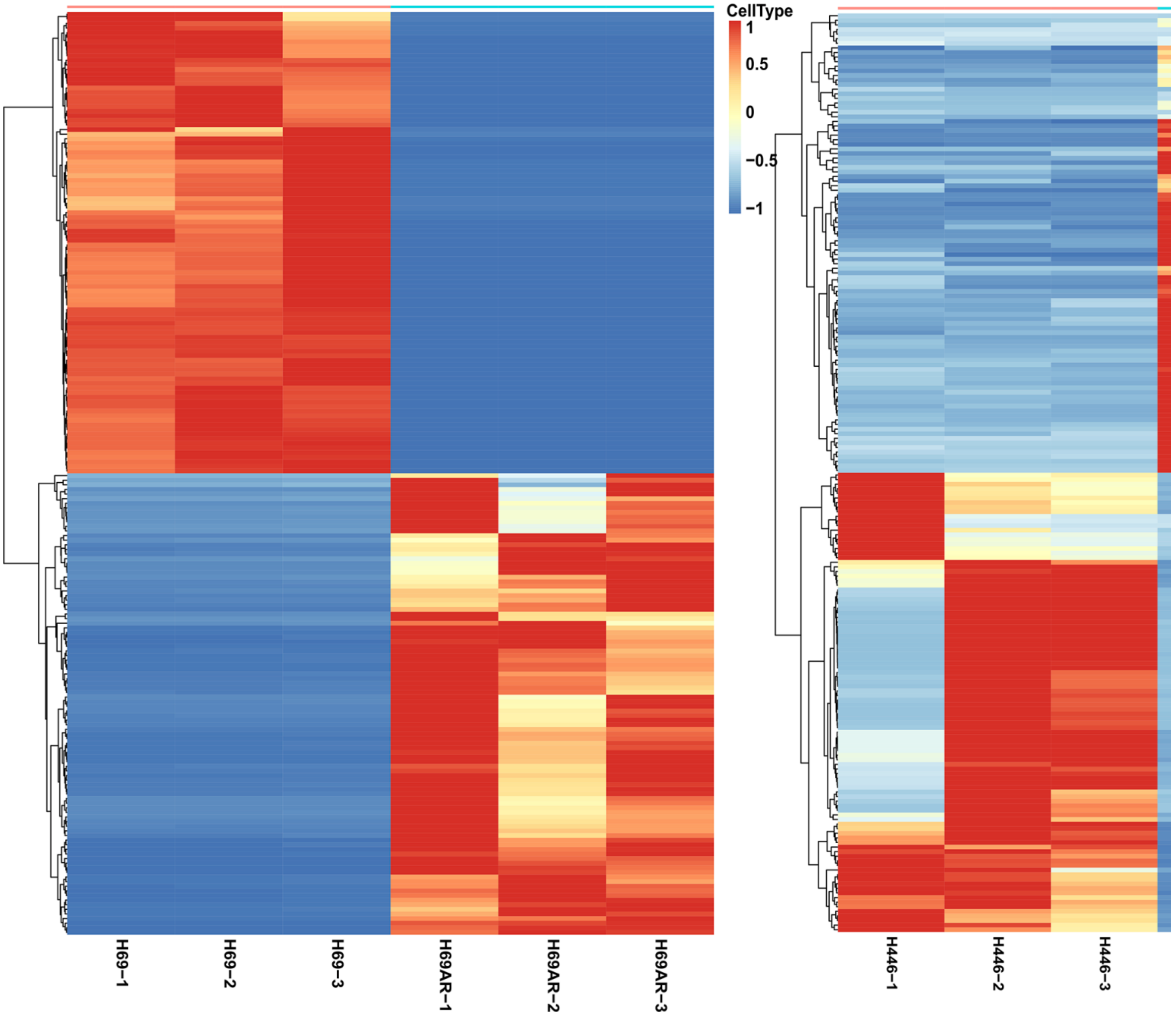

Supplementary Figure 4. (**A**) Heatmap of differentially expressed genes (DEG) in H69 cell lines. The columns show 6 SCLC cell lines including H69-1, H69-2, H69-3, H69AR-1, H69AR-2, and H69AR-3; while the rows show scaled expression (FC < 2/3 or FC > 3/2; P <0.05). (**B**) Heatmap of DEGs in H446 cell lines. The columns show six SCLC cell lines including H446-1, H446-2, H446-3, H446DDP-1, H446DDP-2, and H446DDP-3; while the rows show scaled expression (FC ≥ 3/2 or FC ≤ 2/3; P <0.05).
