# Supplementary material for: DNAH10 mutation correlates with cisplatin sensitivity and tumor mutation burden in small-cell lung cancer
Source: Aging (Albany NY). 2020 Jan 20;12(2):1285–303. doi: 10.18632/aging.102683 (PMC7053592; doi:10.18632/aging.102683)
Supplement: Supplementary Tables [file aging-12-102683-s007..pdf]

SUPPLEMENTARY TABLES

Please browse Full Text version to see the data of Supplementary Tables 1–8

Supplementary Table1 . Characteristics of 55 small cell lung cancer cell lines (SCLC) in GDSC database.

Supplementary Table 2. P-values of Wilcoxon signed rank test (two-tailed) between IC50 values of cisplatin and gene mutations in SCLC cell lines.

Supplementary Table 3. Univariate Cox regression analysis of overall survival (OS) in patients with SCLC.

Supplementary Table 4. DNAH10 mutations discovered in 110 human SCLC specimens (reported by George et al.) and GDSC-SCLC cell lines.

Supplementary Table 5. Sixty upregulated and 97 downregulated DEGs were observed in cisplatin-resistant GDSC-SCLC cell lines.

Supplementary Table 6. Score of single sample gene-set enrichment analysis (ssGSEA) of 55 GDSC-SCLC cell lines.

Supplementary Table 7. Mann-Whitney U test of the score of single sample gene-set enrichment analysis (ssGSEA) between DNAH10-mutated and wild-type DNAH10 cell lines.

Supplementary Table 8. Three other drugs sensitivity between DNAH10-mutated and wild-type DNAH10 cell lines.
