# Supplementary material for: DNAH10 mutation correlates with cisplatin sensitivity and tumor mutation burden in small-cell lung cancer
Source: Aging (Albany NY). 2020 Jan 20;12(2):1285–303. doi: 10.18632/aging.102683 (PMC7053592; doi:10.18632/aging.102683)
Supplement: Supplementary Table 1 [file aging-12-102683-s004..docx]

Supplementary Table1 . Characteristics of 55 small cell lung cancer cell lines (SCLC) in GDSC database

| Num | Cell_line | TCGA classification | Tissue | Tissue sub-type | LN_IC50 | AUC | WES | CNA | Gene Expression | Methylation | Drug Response | MSI | Screen Medium | Growth Properties |
| --- | --- | --- | --- | --- | --- | --- | --- | --- | --- | --- | --- | --- | --- | --- |
| 1 | COLO-668 | SCLC | lung | lung_small_cell_carcinoma | 3.59554 | 0.954294 | Y | Y | Y | Y | Y | MSS/MSI-L | R | Adherent |
| 2 | COR-L279 | SCLC | lung | lung_small_cell_carcinoma | 1.52204 | 0.811194 | Y | Y | Y | Y | Y | MSS/MSI-L | R | Suspension |
| 3 | COR-L311 | SCLC | lung | lung_small_cell_carcinoma | 0.55967 | 0.803309 | Y | Y | Y | Y | Y | MSS/MSI-L | R | Suspension |
| 4 | COR-L32 | SCLC | lung | lung_small_cell_carcinoma | 1.64683 | 0.864816 | Y | Y | Y | Y | Y | MSS/MSI-L | R | Suspension |
| 5 | COR-L88 | SCLC | lung | lung_small_cell_carcinoma | 5.22912 | 0.965927 | Y | Y | Y | Y | Y | MSS/MSI-L | R | Adherent |
| 6 | CPC-N | SCLC | lung | lung_small_cell_carcinoma | 2.83912 | 0.920792 | Y | Y | Y | Y | Y | MSS/MSI-L | R | Suspension |
| 7 | DMS-114 | SCLC | lung | lung_small_cell_carcinoma | 2.80819 | 0.896153 | Y | Y | Y | Y | Y | MSS/MSI-L | D/F12 | Adherent |
| 8 | DMS-273 | SCLC | lung | lung_small_cell_carcinoma | 2.24809 | 0.860454 | Y | Y | Y | Y | Y | MSS/MSI-L | D/F12 | Adherent |
| 9 | DMS-53 | SCLC | lung | lung_small_cell_carcinoma | 5.17527 | 0.984168 | Y | Y | Y | Y | Y | MSS/MSI-L | D/F12 | Adherent |
| 10 | IST-SL1 | SCLC | lung | lung_small_cell_carcinoma | 3.53665 | 0.949787 | Y | Y | Y | Y | Y | MSS/MSI-L | R | Semi-Adherent |
| 11 | IST-SL2 | SCLC | lung | lung_small_cell_carcinoma | 3.31862 | 0.942043 | Y | Y | Y | Y | Y | MSS/MSI-L | R | Suspension |
| 12 | LB647-SCLC | SCLC | lung | lung_small_cell_carcinoma | 2.38878 | 0.901355 | Y | Y | Y | Y | Y | MSS/MSI-L | R | Semi-Adherent |
| 13 | LU-134-A | SCLC | lung | lung_small_cell_carcinoma | 2.90622 | 0.86316 | Y | Y | Y | Y | Y | MSS/MSI-L | R | Suspension |
| 14 | LU-135 | SCLC | lung | lung_small_cell_carcinoma | 2.3611 | 0.901014 | Y | Y | Y | Y | Y | MSS/MSI-L | R | Suspension |
| 15 | LU-139 | SCLC | lung | lung_small_cell_carcinoma | 3.94166 | 0.928845 | Y | Y | Y | Y | Y | MSS/MSI-L | R | Suspension |
| 16 | LU-165 | SCLC | lung | lung_small_cell_carcinoma | 5.23329 | 0.981668 | Y | Y | Y | Y | Y | MSS/MSI-L | R | Suspension |
| 17 | MS-1 | SCLC | lung | lung_small_cell_carcinoma | 4.4174 | 0.944739 | Y | Y | Y | Y | Y | MSS/MSI-L | R | Suspension |
| 18 | NCI-H1048 | SCLC | lung | lung_small_cell_carcinoma | 2.31372 | 0.896933 | Y | Y | Y | Y | Y | MSI-H | D/F12 | Adherent |
| 19 | NCI-H1092 | SCLC | lung | lung_small_cell_carcinoma | 5.12634 | 0.943237 | Y | Y | Y | Y | Y | MSS/MSI-L | D/F12 | Suspension |
| 20 | NCI-H1105 | SCLC | lung | lung_small_cell_carcinoma | 1.4164 | 0.803526 | Y | Y | Y | Y | Y | MSS/MSI-L | D/F12 | Suspension |
| 21 | NCI-H1341 | SCLC | lung | lung_small_cell_carcinoma | 1.73231 | 0.844068 | Y | Y | Y | Y | Y | MSS/MSI-L | D/F12 | Suspension |
| 22 | NCI-H1417 | SCLC | lung | lung_small_cell_carcinoma | 0.60579 | 0.724176 | Y | Y | Y | Y | Y | MSS/MSI-L | R | Suspension |
| 23 | NCI-H1436 | SCLC | lung | lung_small_cell_carcinoma | 4.96737 | 0.98442 | Y | Y | Y | Y | Y | MSS/MSI-L | D/F12 | Suspension |
| 24 | NCI-H146 | SCLC | lung | lung_small_cell_carcinoma | 3.13209 | 0.982625 | Y | Y | Y | Y | Y | MSS/MSI-L | R | Suspension |
| 25 | NCI-H1688 | SCLC | lung | lung_small_cell_carcinoma | 3.12163 | 0.925357 | Y | Y | Y | Y | Y | MSS/MSI-L | R | Adherent |
| 26 | NCI-H1694 | SCLC | lung | lung_small_cell_carcinoma | 2.33257 | 0.878929 | Y | Y | Y | Y | Y | MSS/MSI-L | D/F12 | Suspension |
| 27 | NCI-H1836 | SCLC | lung | lung_small_cell_carcinoma | 5.00826 | 0.981995 | Y | Y | Y | Y | Y | MSS/MSI-L | D/F12 | Suspension |
| 28 | NCI-H187 | SCLC | lung | lung_small_cell_carcinoma | 3.33924 | 0.934052 | Y | Y | Y | Y | Y | MSS/MSI-L | R | Suspension |
| 29 | NCI-H1876 | SCLC | lung | lung_small_cell_carcinoma | -0.00328 | 0.574847 | Y | Y | Y | Y | Y | MSS/MSI-L | D/F12 | Adherent |
| 30 | NCI-H196 | SCLC | lung | lung_small_cell_carcinoma | 7.61708 | 0.85659 | Y | Y | Y | Y | Y | MSS/MSI-L | R | Adherent |
| 31 | NCI-H1963 | SCLC | lung | lung_small_cell_carcinoma | 3.22428 | 0.959348 | Y | Y | Y | Y | Y | MSS/MSI-L | R | Suspension |
| 32 | NCI-H2029 | SCLC | lung | lung_small_cell_carcinoma | 4.79723 | 0.990779 | Y | Y | Y | Y | Y | MSS/MSI-L | D/F12 | Adherent |
| 33 | NCI-H2066 | SCLC | lung | lung_small_cell_carcinoma | 6.67234 | 0.945166 | Y | Y | Y | Y | Y | MSS/MSI-L | D/F12 | Adherent |
| 34 | NCI-H209 | SCLC | lung | lung_small_cell_carcinoma | -2.11309 | 0.544441 | Y | Y | Y | Y | Y | MSS/MSI-L | R | Suspension |
| 35 | NCI-H211 | SCLC | lung | lung_small_cell_carcinoma | 3.24061 | 0.91748 | Y | Y | Y | Y | Y | MSS/MSI-L | R | Adherent |
| 36 | NCI-H2141 | SCLC | lung | lung_small_cell_carcinoma | 3.84884 | 0.967798 | Y | Y | Y | Y | Y | MSS/MSI-L | D/F12 | Suspension |
| 37 | NCI-H2227 | SCLC | lung | lung_small_cell_carcinoma | 2.66576 | 0.887788 | Y | Y | Y | Y | Y | MSS/MSI-L | R | Semi-Adherent |
| 38 | NCI-H345 | SCLC | lung | lung_small_cell_carcinoma | 4.50882 | 0.976243 | Y | Y | Y | Y | Y | MSS/MSI-L | D/F12 | Suspension |
| 39 | NCI-H446 | SCLC | lung | lung_small_cell_carcinoma | 4.0806 | 0.956586 | Y | Y | Y | Y | Y | MSS/MSI-L | R | Semi-Adherent |
| 40 | NCI-H510A | SCLC | lung | lung_small_cell_carcinoma | 2.70465 | 0.841264 | Y | Y | Y | Y | Y | MSS/MSI-L | D/F12 | Semi-Adherent |
| 41 | NCI-H524 | SCLC | lung | lung_small_cell_carcinoma | 1.94925 | 0.856613 | Y | Y | Y | Y | Y | MSS/MSI-L | R | Suspension |
| 42 | NCI-H526 | SCLC | lung | lung_small_cell_carcinoma | 1.45885 | 0.74644 | Y | Y | Y | Y | Y | MSS/MSI-L | R | Suspension |
| 43 | NCI-H64 | SCLC | lung | lung_small_cell_carcinoma | 2.72538 | 0.983137 | Y | Y | Y | Y | Y | MSS/MSI-L | R | Suspension |
| 44 | NCI-H69 | SCLC | lung | lung_small_cell_carcinoma | 4.36538 | 0.972378 | Y | Y | Y | Y | Y | MSS/MSI-L | R | Suspension |
| 45 | NCI-H82 | SCLC | lung | lung_small_cell_carcinoma | 1.5137 | 0.866267 | Y | Y | Y | Y | Y | MSS/MSI-L | R | Suspension |
| 46 | NCI-H841 | SCLC | lung | lung_small_cell_carcinoma | 2.48157 | 0.891806 | Y | Y | Y | Y | Y | MSS/MSI-L | D/F12 | Adherent |
| 47 | NCI-H847 | SCLC | lung | lung_small_cell_carcinoma | 4.1933 | 0.991174 | Y | Y | Y | Y | Y | MSS/MSI-L | D/F12 | Adherent |
| 48 | SBC-1 | SCLC | lung | lung_small_cell_carcinoma | 4.66877 | 0.943041 | Y | Y | Y | Y | Y | MSS/MSI-L | R | Suspension |
| 49 | SBC-3 | SCLC | lung | lung_small_cell_carcinoma | 1.24655 | 0.771869 | Y | Y | Y | Y | Y | MSS/MSI-L | D/F12 | Adherent |
| 50 | SBC-5 | SCLC | lung | lung_small_cell_carcinoma | 3.07504 | 0.962196 | Y | Y | Y | Y | Y | MSS/MSI-L | D/F12 | Adherent |
| 51 | DMS-79 | SCLC | lung | lung_small_cell_carcinoma | 3.10091 | 0.898847 | Y | Y | Y | Y | Y | NA | R | Suspension |
| 52 | COR-L95 | SCLC | lung | lung_small_cell_carcinoma | 3.12261 | 0.91559 | Y | Y | Y | N | Y | MSS/MSI-L | R | Adherent |
| 53 | NCI-H1304 | SCLC | lung | lung_small_cell_carcinoma | 4.00399 | 0.902256 | Y | Y | Y | N | Y | MSS/MSI-L | D/F12 | Suspension |
| 54 | SHP-77 | SCLC | lung | lung_small_cell_carcinoma | 4.67977 | 0.990833 | Y | Y | Y | N | Y | MSS/MSI-L | R | Semi-Adherent |
| 55 | SW1271 | SCLC | lung | lung_small_cell_carcinoma | 3.65971 | 0.941666 | Y | Y | Y | N | Y | MSS/MSI-L | D/F12 | Adherent |

WES: Whole Exome Sequencing; CNA: Copy Number Alterations; MSI: Microsatellite instability Status; IC50: The half maximal inhibitory concentration; Y: yes; N: no; NA: not available; R: RPMI; D/F12: DMEM/F12; MSS: microsatellite stable; MSI-L: microsatellite instability low; MSI-H: microsatellite instability high; GDSC: Genomics of Drug Sensitivity in Cancer.
