# Supplementary material for: DNAH10 mutation correlates with cisplatin sensitivity and tumor mutation burden in small-cell lung cancer
Source: Aging (Albany NY). 2020 Jan 20;12(2):1285–303. doi: 10.18632/aging.102683 (PMC7053592; doi:10.18632/aging.102683)
Supplement: Supplementary Table 2 [file aging-12-102683-s003..docx]

S Table 2. P-values of Wilcoxon signed rank test (two-tailed) between IC50 values of Cisplatin and gene mutations in SCLC cell lines.

| Gene | P.value |
| --- | --- |
| EYS | 0.000247321 |
| WDR87 | 0.000731398 |
| ACAN | 0.000877961 |
| KDR | 0.008889027 |
| LRP1 | 0.008889027 |
| RELN | 0.011919976 |
| PLEC | 0.014005055 |
| FAT1 | 0.017051105 |
| MYO9A | 0.019658033 |
| DNAH8 | 0.026569554 |
| WRN | 0.028769111 |
| AIM1 | 0.032840175 |
| EP400 | 0.033948708 |
| DNAH10 | 0.034970118 |
| CEP350 | 0.040187954 |
| TAF1L | 0.040273102 |
| OR8K1 | 0.042911869 |
| ZEB1 | 0.042911869 |
| TP53 | 0.04526722 |
| FSTL5 | 0.0457816 |
| MUC2 | 0.047509999 |
| CDH20 | 0.048517826 |
| TSPEAR | 0.051948357 |
| COL6A6 | 0.055320514 |
| MYO18B | 0.061650089 |
| SAMD9L | 0.063394502 |
| MUC17 | 0.06785446 |
| AC007731.1 | 0.07041029 |
| ARAP2 | 0.072050894 |
| SORCS3 | 0.074642625 |
| PTPRB | 0.07917396 |
| AKAP6 | 0.081589945 |
| CNTRL | 0.081589945 |
| FAT4 | 0.083015353 |
| CD163 | 0.083521241 |
| DSG1 | 0.086705974 |
| ZNF208 | 0.088051546 |
| PPP1R9A | 0.088531668 |
| ZIC1 | 0.088531668 |
| DNAH2 | 0.090354785 |
| ANKFN1 | 0.103524031 |
| ERBB4 | 0.109638966 |
| FAM5B | 0.109638966 |
| NLRP5 | 0.109672855 |
| SCN1A | 0.110031362 |
| NIPBL | 0.115973399 |
| ATRNL1 | 0.116020687 |
| SCN7A | 0.116020687 |
| EML5 | 0.122671882 |
| TRIO | 0.122671882 |
| SYNE1 | 0.125936785 |
| PDE4DIP | 0.128573363 |
| SHROOM3 | 0.128573363 |
| VWA3B | 0.129601936 |
| CACNA1C | 0.131413486 |
| NLRP3 | 0.131413486 |
| CACNA1H | 0.135240879 |
| HFM1 | 0.13681271 |
| KCNH7 | 0.13681271 |
| MGAM | 0.137788218 |
| ABCA6 | 0.144313381 |
| RGPD4 | 0.144313381 |
| MYPN | 0.152105672 |
| RBBP6 | 0.152105672 |
| CSMD3 | 0.155126296 |
| MYH15 | 0.156766711 |
| RYR2 | 0.15931819 |
| OAS3 | 0.160198071 |
| PTPRU | 0.16859168 |
| STON2 | 0.16859168 |
| LRP1B | 0.17136753 |
| FREM1 | 0.172429046 |
| EYA1 | 0.177295054 |
| ENSG00000250423 | 0.180665446 |
| CDH18 | 0.180903065 |
| ENSG00000121031 | 0.182447371 |
| OR6F1 | 0.186308194 |
| KIF2B | 0.186487008 |
| LAMA5 | 0.186487008 |
| MUC5B | 0.188607582 |
| HYDIN | 0.195870388 |
| ABCA8 | 0.197208758 |
| ACSM5 | 0.197208758 |
| NAV3 | 0.197360347 |
| ABCA13 | 0.201278099 |
| CNGB3 | 0.205287641 |
| HSPG2 | 0.207034963 |
| MCF2 | 0.207034963 |
| XIRP2 | 0.21131257 |
| LPA | 0.215260778 |
| SIGLEC12 | 0.215260778 |
| TRPA1 | 0.215260778 |
| CACHD1 | 0.22555741 |
| CARD11 | 0.22555741 |
| GRIN3A | 0.22555741 |
| CDH8 | 0.226027804 |
| DNAH14 | 0.229878057 |
| ADGB | 0.232855274 |
| FCGBP | 0.233825519 |
| KIAA1109 | 0.235955664 |
| DNAH6 | 0.236184504 |
| ANKRD30A | 0.245939619 |
| DNAH17 | 0.247139576 |
| PTEN | 0.247139576 |
| ANK2 | 0.255449182 |
| PCDH15 | 0.256287181 |
| BRIP1 | 0.258428975 |
| LPHN3 | 0.258428975 |
| PTPN13 | 0.258428975 |
| PXDNL | 0.267485579 |
| SRRM2 | 0.270049733 |
| NID2 | 0.272654078 |
| ABCB1 | 0.282007025 |
| ZFPM2 | 0.282007025 |
| HERC2 | 0.283262736 |
| C6 | 0.289973566 |
| CREBBP | 0.289973566 |
| FAM5C | 0.292428757 |
| SI | 0.294136048 |
| CPS1 | 0.294296918 |
| LINGO2 | 0.294296918 |
| CNTNAP4 | 0.295519042 |
| ENSG00000188219 | 0.295519042 |
| KIAA1239 | 0.301660101 |
| UNC80 | 0.305006502 |
| ZNF479 | 0.306158816 |
| PTPRZ1 | 0.30692431 |
| ZFHX4 | 0.308973489 |
| PCDH11X | 0.313641332 |
| TYR | 0.313641332 |
| LAMA3 | 0.317040452 |
| MYH9 | 0.319883821 |
| PCDHG_cluster | 0.319883821 |
| NBPF10 | 0.321207438 |
| KIAA1211 | 0.325917535 |
| DNAH3 | 0.328347182 |
| ASTN2 | 0.33317952 |
| UTP20 | 0.33317952 |
| MORC1 | 0.33848742 |
| LAMA2 | 0.343328212 |
| MUC6 | 0.346805475 |
| NELL1 | 0.346805475 |
| SZT2 | 0.346805475 |
| ZNF462 | 0.346805475 |
| SLC8A3 | 0.351132376 |
| COL24A1 | 0.351350502 |
| TEP1 | 0.351350502 |
| FAM135B | 0.351762057 |
| POLQ | 0.35247606 |
| KIAA1671 | 0.360764514 |
| NRXN1 | 0.360764514 |
| POM121L12 | 0.360764514 |
| SAMD9 | 0.360764514 |
| SELP | 0.360764514 |
| MYH1 | 0.364504752 |
| MUC12 | 0.370886383 |
| PHKB | 0.375049669 |
| ST18 | 0.375049669 |
| CSMD1 | 0.382525689 |
| CACNA1E | 0.389663354 |
| POTEH | 0.390630452 |
| TRHDE | 0.391680506 |
| COL22A1 | 0.402601749 |
| OR9G4 | 0.404597085 |
| COL11A1 | 0.404925109 |
| VPS13B | 0.41410352 |
| TNXB | 0.415585574 |
| ANKRD12 | 0.419852447 |
| OR5T2 | 0.419852447 |
| SF3B2 | 0.419852447 |
| DNAH7 | 0.431093448 |
| NAV2 | 0.434573432 |
| SIGLEC10 | 0.434573432 |
| BOD1L | 0.435420404 |
| MYH13 | 0.435420404 |
| SCAND3 | 0.435420404 |
| SPEG | 0.435420404 |
| VCAN | 0.435420404 |
| OBSCN | 0.438828521 |
| TG | 0.43897943 |
| USH2A | 0.439597557 |
| ANKRD30B | 0.449425794 |
| NLRP4 | 0.449425794 |
| SPAG17 | 0.449425794 |
| TCHH | 0.449425794 |
| AC027369_8 | 0.451301162 |
| BCLAF1 | 0.451301162 |
| NCAM2 | 0.451301162 |
| SDK1 | 0.451301162 |
| SYTL2 | 0.451301162 |
| ALMS1 | 0.459315705 |
| DCHS1 | 0.459315705 |
| TTN | 0.46196106 |
| C12orf51 | 0.464549534 |
| CYP11B1 | 0.464549534 |
| IGF2R | 0.464549534 |
| CTNNA2 | 0.466142997 |
| DSEL | 0.467484648 |
| GRM1 | 0.473790732 |
| MYH7 | 0.479939486 |
| DOCK2 | 0.480054952 |
| PKHD1 | 0.480054952 |
| KCNK10 | 0.483970724 |
| TMEM132D | 0.485006467 |
| NEB | 0.486234799 |
| KALRN | 0.488503614 |
| MDN1 | 0.495591522 |
| DST | 0.498908078 |
| CSMD2 | 0.500747801 |
| SAGE1 | 0.500747801 |
| CDH10 | 0.503449416 |
| AHNAK | 0.508521584 |
| COL6A3 | 0.508521584 |
| MACF1 | 0.51147847 |
| CDK5RAP2 | 0.517814843 |
| LRFN5 | 0.517814843 |
| SLC35F4 | 0.517814843 |
| TAF15 | 0.517814843 |
| FLG | 0.51845386 |
| C12orf35 | 0.518624229 |
| SYNE2 | 0.523068389 |
| TPTE | 0.534022502 |
| COL12A1 | 0.534267615 |
| LRRK2 | 0.534267615 |
| DISP2 | 0.535159777 |
| PCDHB7 | 0.535159777 |
| UBR4 | 0.537818439 |
| BAI3 | 0.544063508 |
| OR11H12 | 0.544063508 |
| COL14A1 | 0.549639733 |
| DMD | 0.549639733 |
| LRRC7 | 0.552767295 |
| CA2 | 0.552780257 |
| HDAC9 | 0.552780257 |
| HMCN1 | 0.555573534 |
| ABCC12 | 0.560706774 |
| DYSF | 0.565469775 |
| THSD7A | 0.565469775 |
| BAZ2B | 0.570663245 |
| MYH8 | 0.570663245 |
| NLRP12 | 0.577581678 |
| VWF | 0.577581678 |
| FRAS1 | 0.583241683 |
| CUBN | 0.583317196 |
| SVEP1 | 0.583317196 |
| C10orf90 | 0.588806049 |
| FCRL3 | 0.588806049 |
| MUC16 | 0.591330284 |
| APOB | 0.5937049 |
| GRM5 | 0.594681924 |
| OR10J1 | 0.594681924 |
| POTEC | 0.594681924 |
| ZEB2 | 0.594681924 |
| HCN1 | 0.598214002 |
| MLL3 | 0.598214002 |
| OR8H3 | 0.598756879 |
| USP34 | 0.598756879 |
| ATM | 0.607194182 |
| FREM3 | 0.607194182 |
| TPO | 0.607194182 |
| MTUS2 | 0.614180505 |
| C1orf173 | 0.617513792 |
| RB1 | 0.624948181 |
| ZNF536 | 0.625634344 |
| ITGAD | 0.628497276 |
| ZNF729 | 0.628497276 |
| ADAMTS20 | 0.633400513 |
| ROS1 | 0.636245927 |
| OTOG | 0.643873375 |
| UNC13C | 0.643873375 |
| GRIA2 | 0.644681253 |
| MKI67 | 0.644681253 |
| MYH11 | 0.644681253 |
| PLCE1 | 0.644681253 |
| ADAMTS12 | 0.646347768 |
| EP300 | 0.646347768 |
| FMN2 | 0.646347768 |
| CENPF | 0.647256554 |
| CNTN5 | 0.647256554 |
| GRIP1 | 0.647256554 |
| NOTCH1 | 0.647256554 |
| PDGFRA | 0.647256554 |
| PLCH1 | 0.647256554 |
| CMYA5 | 0.647606969 |
| AHNAK2 | 0.650699209 |
| DNAH5 | 0.662539846 |
| PCNT | 0.663760598 |
| LAMA1 | 0.664585302 |
| AKAP13 | 0.665179792 |
| C5orf42 | 0.665179792 |
| MYO7B | 0.665179792 |
| RYR3 | 0.665281502 |
| LRP2 | 0.667242494 |
| ZIM2 | 0.670485015 |
| HEATR7B2 | 0.675067308 |
| ADAM2 | 0.678885751 |
| PKD1L2 | 0.683046567 |
| PCLO | 0.685702518 |
| MUC4 | 0.694281248 |
| FLNC | 0.699036111 |
| PRDM9 | 0.701571616 |
| TRPM2 | 0.701571616 |
| GPRC6A | 0.702534264 |
| GRIN2B | 0.702534264 |
| THSD7B | 0.702534264 |
| ADAMTSL1 | 0.712012198 |
| ABCC11 | 0.716493177 |
| OR14K1 | 0.716493177 |
| C10orf112 | 0.720022097 |
| DPP6 | 0.720022097 |
| COL19A1 | 0.722206717 |
| CRB1 | 0.728778967 |
| DSCAML1 | 0.732081971 |
| MLL2 | 0.738153603 |
| ANKRD55 | 0.738630199 |
| SPTA1 | 0.739057849 |
| CCDC108 | 0.742058336 |
| COL8A1 | 0.742058336 |
| MUC19 | 0.743538578 |
| ABCB5 | 0.751829712 |
| HRNR | 0.755212107 |
| ACSM2B | 0.757385478 |
| IGSF3 | 0.757385478 |
| FLG2 | 0.762072014 |
| OVCH1 | 0.762072014 |
| WBSCR17 | 0.762072014 |
| TNN | 0.762683969 |
| CNTNAP5 | 0.763550816 |
| SACS | 0.769692484 |
| STAB2 | 0.769692484 |
| ANKS1B | 0.776278747 |
| C10orf71 | 0.782241057 |
| C20orf26 | 0.782241057 |
| LRRIQ1 | 0.782241057 |
| OR4C16 | 0.782241057 |
| RTTN | 0.782241057 |
| COL3A1 | 0.787673965 |
| MYH4 | 0.787673965 |
| ZNF99 | 0.787673965 |
| C7orf58 | 0.795299026 |
| GPR112 | 0.797034547 |
| DNAH9 | 0.800771464 |
| ZNF521 | 0.802547735 |
| FSCB | 0.805764614 |
| KCNU1 | 0.805764614 |
| ADCY8 | 0.814436706 |
| SRCAP | 0.814436706 |
| RYR1 | 0.821528876 |
| SETD2 | 0.822985356 |
| SORL1 | 0.822985356 |
| PKHD1L1 | 0.83176909 |
| PAPPA2 | 0.837220459 |
| DCC | 0.842238861 |
| WNK3 | 0.843535086 |
| FBN2 | 0.849260554 |
| ZSCAN20 | 0.853020355 |
| CLSTN2 | 0.864189819 |
| PAPPA | 0.864189819 |
| TFAP2D | 0.864189819 |
| DCHS2 | 0.87244466 |
| DPP10 | 0.87244466 |
| OR52R1 | 0.87244466 |
| UTRN | 0.879041769 |
| FAT3 | 0.882839962 |
| CD163L1 | 0.884930859 |
| UBQLN3 | 0.884930859 |
| ZP4 | 0.884930859 |
| CCDC141 | 0.891943159 |
| FAM75D4 | 0.891943159 |
| OR6Y1 | 0.891943159 |
| NCKAP1L | 0.905750133 |
| SPTB | 0.905750133 |
| TRIM58 | 0.905750133 |
| MAGEC1 | 0.911503896 |
| KIAA0947 | 0.922999649 |
| ALPK3 | 0.926628601 |
| DOCK7 | 0.926628601 |
| LYST | 0.926628601 |
| TKTL2 | 0.926628601 |
| USP32 | 0.931116406 |
| ALPK2 | 0.947558536 |
| ASPM | 0.947558536 |
| CLVS2 | 0.947558536 |
| LRRK1 | 0.947558536 |
| POTEG | 0.947558536 |
| ZNF142 | 0.947558536 |
| ODZ3 | 0.950768615 |
| XDH | 0.950768615 |
| ABCA12 | 0.95333035 |
| RIMS2 | 0.95333035 |
| TRRAP | 0.957177427 |
| MSH4 | 0.968520068 |
| OR10Q1 | 0.968520068 |
| SHANK2 | 0.968520068 |
| BSN | 0.970449839 |
| CDH19 | 0.970449839 |
| COL11A2 | 0.970449839 |
| SMARCA4 | 0.970449839 |
| COL5A2 | 0.989505264 |
| OR5L2 | 0.989505264 |
| TNKS | 0.989505264 |
| FBXO10 | 0.989505264 |
| ZNF804B | 0.990147896 |
| DDX12 | 0.990661072 |
| GPR98 | 0.992382493 |
| SPHKAP | 0.992382493 |
| PKD1L1 | 1 |
| DNAH11 | 1 |
| ABCA9 | 1 |
| ODZ1 | 1 |
| TNR | 1 |
| RP1L1 | 1 |
| SLC5A10 | 1 |
| KIAA0240 | 1 |
