# Supplementary material for: DNAH10 mutation correlates with cisplatin sensitivity and tumor mutation burden in small-cell lung cancer
Source: Aging (Albany NY). 2020 Jan 20;12(2):1285–303. doi: 10.18632/aging.102683 (PMC7053592; doi:10.18632/aging.102683)
Supplement: Supplementary Table 3 [file aging-12-102683-s005..docx]

S Table 3. Univariate Cox regression analysis of overall survival (OS) in patients with SCLC.

| gene | HR | z | pvalue |
| --- | --- | --- | --- |
| NAALAD2 | 5.852138281 | 3.881438731 | 0.00010384 |
| DNAH10 | 4.891947968 | 3.577892936 | 0.000346375 |
| UTRN | 2.822160974 | 3.048107574 | 0.002302875 |
| WDR87 | 2.882066754 | 3.00153341 | 0.002686236 |
| OR4A5 | 3.365352408 | 2.797723781 | 0.00514641 |
| MNDA | 3.136924992 | 2.779756577 | 0.005439966 |
| VPS13D | 3.143942256 | 2.767814359 | 0.005643359 |
| LTN1 | 3.067150098 | 2.765074799 | 0.005690974 |
| PCDHB13 | 3.289049011 | 2.71501761 | 0.006627225 |
| BNC2 | 3.184210194 | 2.66025122 | 0.007808239 |
| FCRL4 | 2.482181655 | 2.491500858 | 0.012720466 |
| SSFA2 | 2.756216058 | 2.478736736 | 0.013184858 |
| GRIN2B | 2.914509199 | 2.447130245 | 0.014399879 |
| PIEZO2 | 2.10187489 | 2.35466627 | 0.018539348 |
| DST | 2.241769336 | 2.331419844 | 0.019731232 |
| CHD8 | 0.096911916 | -2.31399957 | 0.020667746 |
| GPR50 | 2.706147158 | 2.268013297 | 0.023328396 |
| BOD1L | 2.254598452 | 2.255046584 | 0.024130405 |
| NBEA | 2.469482068 | 2.227980473 | 0.025881817 |
| SLC4A10 | 2.216910296 | 2.20929683 | 0.027154002 |
| LTBP4 | 2.596947643 | 2.203953697 | 0.027527601 |
| CCT8L2 | 2.579058827 | 2.182791321 | 0.029051181 |
| MMRN1 | 2.776890176 | 2.169716243 | 0.030028349 |
| COL6A3 | 2.551687594 | 2.168121305 | 0.030149458 |
| LRRTM4 | 2.279326716 | 2.163028201 | 0.030539011 |
| AGRN | 2.526658738 | 2.144487268 | 0.031993864 |
| MED12 | 2.338156613 | 2.120275009 | 0.03398286 |
| ABL2 | 2.674253735 | 2.101187802 | 0.035624484 |
| CEP128 | 2.482539275 | 2.101033856 | 0.035637994 |
| NYAP2 | 2.487000285 | 2.082551296 | 0.037292143 |
| CAMSAP1 | 0.227413874 | -2.058546353 | 0.039537716 |
| ACACB | 2.625149906 | 2.052627757 | 0.040108693 |
| ANKRD30B | 1.798079501 | 2.027241792 | 0.042637691 |
| RSF1 | 2.398570284 | 2.020361463 | 0.043345908 |
| THSD7B | 1.890726942 | 1.98000822 | 0.047702605 |
| ZNF536 | 1.888539129 | 1.976061571 | 0.048147804 |
| PKD1L1 | 2.337541473 | 1.969910776 | 0.048848597 |
| OTOF | 2.046564249 | 1.952975107 | 0.050822545 |
| SALL3 | 1.899865022 | 1.936812769 | 0.052768234 |
| SLC17A6 | 2.087478493 | 1.933987281 | 0.053114692 |
| MXRA5 | 0.142510427 | -1.931597772 | 0.053409171 |
| FGA | 2.175920487 | 1.930751484 | 0.053513793 |
| UNC13A | 0.408745401 | -1.920432278 | 0.05480332 |
| MLL3 | 2.176955538 | 1.912235001 | 0.055846059 |
| CREBBP | 1.957087388 | 1.865764042 | 0.062074385 |
| CRB1 | 2.025765892 | 1.846800705 | 0.06477603 |
| CCNB3 | 0.155785435 | -1.844028172 | 0.065179035 |
| VWDE | 0.266401269 | -1.840615028 | 0.065677994 |
| RBP3 | 0.266675403 | -1.838261434 | 0.066023891 |
| MYH4 | 0.156949299 | -1.836055889 | 0.066349391 |
| SORCS1 | 1.949825707 | 1.835048687 | 0.066498476 |
| OR4K2 | 0.267342447 | -1.833655045 | 0.066705216 |
| TECPR2 | 2.188429643 | 1.818539599 | 0.0689817 |
| JPH2 | 2.363165125 | 1.80826617 | 0.070565084 |
| TLR8 | 0.273612551 | -1.802801255 | 0.071419433 |
| KCNG2 | 2.314036453 | 1.774539436 | 0.075973965 |
| CSMD2 | 1.686611001 | 1.760457006 | 0.078330351 |
| RP1 | 1.795563906 | 1.752059301 | 0.079763612 |
| GPRC6A | 1.79036078 | 1.736764079 | 0.082428822 |
| TLN1 | 0.174149358 | -1.733743533 | 0.082963599 |
| SPTBN4 | 2.015247692 | 1.723311544 | 0.084832201 |
| GPR139 | 0.361711524 | -1.715070067 | 0.086332382 |
| MYH2 | 0.480989366 | -1.70758118 | 0.087714083 |
| TBX22 | 0.366561495 | -1.695916063 | 0.089901777 |
| OR4N2 | 2.070683792 | 1.690710695 | 0.090892071 |
| LPPR4 | 0.417084257 | -1.687438474 | 0.091519071 |
| TAF1L | 1.785336307 | 1.683569928 | 0.092264814 |
| DYNC1H1 | 1.905945673 | 1.680119372 | 0.092934093 |
| PCDHB7 | 2.065913622 | 1.675151409 | 0.093904528 |
| PPFIA2 | 1.869806671 | 1.655593432 | 0.097804181 |
| SSC5D | 0.305539953 | -1.646947031 | 0.099568934 |
| VPS13B | 0.425259181 | -1.643675177 | 0.100243316 |
| LAMA1 | 1.758434092 | 1.634263975 | 0.102203434 |
| MYO9A | 2.156186536 | 1.627204182 | 0.10369374 |
| OR6B1 | 2.011037997 | 1.626868209 | 0.103765091 |
| POLQ | 0.381900257 | -1.624434433 | 0.104283123 |
| MAGEC1 | 1.711669825 | 1.619687518 | 0.105299419 |
| GRM3 | 1.843163548 | 1.613409517 | 0.106655567 |
| FAM75D1 | 1.783004222 | 1.607555981 | 0.10793246 |
| OR4K17 | 2.125643782 | 1.606019095 | 0.108269714 |
| RGS6 | 0.315316235 | -1.605577508 | 0.108366771 |
| OR4A16 | 1.791358174 | 1.605219684 | 0.108445467 |
| KDR | 0.199346482 | -1.60001092 | 0.109596161 |
| APBA2 | 1.987202862 | 1.593236064 | 0.111107244 |
| ZIC4 | 0.39160551 | -1.582461672 | 0.113544215 |
| NES | 0.44240571 | -1.575736216 | 0.115086608 |
| PHACTR3 | 0.323012465 | -1.569063261 | 0.116633199 |
| OR2L2 | 1.876255488 | 1.563712414 | 0.117885121 |
| CSMD1 | 1.543955959 | 1.558716971 | 0.119063383 |
| UNC80 | 1.623686031 | 1.553839923 | 0.120222605 |
| GLB1L2 | 2.083995792 | 1.551486065 | 0.120785244 |
| TEX15 | 1.868341929 | 1.548589549 | 0.12148042 |
| MAGEC3 | 0.329921823 | -1.540457951 | 0.123448765 |
| HMCN1 | 0.515898127 | -1.53873499 | 0.123869006 |
| COL6A6 | 0.451231158 | -1.53748228 | 0.124175251 |
| PCDH11Y | 1.941302663 | 1.537260376 | 0.124229561 |
| TTN | 1.532400038 | 1.536823972 | 0.124336421 |
| FBN1 | 1.846180483 | 1.527774024 | 0.126568654 |
| ATG2A | 1.921207824 | 1.520949731 | 0.128272453 |
| OBSCN | 1.602029273 | 1.509245648 | 0.131236019 |
| PCDH11X | 1.68368273 | 1.508370287 | 0.131459783 |
| AK5 | 1.90910578 | 1.506868474 | 0.131844374 |
| KIAA1109 | 0.338811798 | -1.506148628 | 0.132029024 |
| UBE2NL | 0.342522081 | -1.491008125 | 0.135959362 |
| ALPK2 | 0.463215257 | -1.489089014 | 0.13646393 |
| WDR52 | 1.8929671 | 1.484208547 | 0.137753603 |
| ZNF676 | 1.896944146 | 1.464248339 | 0.143126107 |
| OR5D18 | 0.3503709 | -1.459543636 | 0.14441554 |
| AKAP6 | 1.801580645 | 1.45657202 | 0.145234556 |
| TRPA1 | 1.793475514 | 1.455604699 | 0.145501928 |
| RREB1 | 0.422705682 | -1.452726001 | 0.146299845 |
| ALPI | 1.864654945 | 1.451954529 | 0.146514249 |
| GAB4 | 0.47320666 | -1.44710023 | 0.147868854 |
| TLR4 | 0.42572984 | -1.442506016 | 0.149159676 |
| NCAN | 1.857377302 | 1.439575321 | 0.149987586 |
| FAM83C | 1.862765183 | 1.4355169 | 0.151139856 |
| STXBP5L | 1.852852207 | 1.434395915 | 0.151459313 |
| ZCCHC16 | 1.953787769 | 1.432854339 | 0.151899469 |
| NLRP13 | 2.09784787 | 1.422715022 | 0.154818784 |
| SELL | 1.952731245 | 1.414165704 | 0.157313255 |
| OR8H2 | 0.363142638 | -1.409307082 | 0.158744386 |
| AHNAK2 | 0.483999515 | -1.404894241 | 0.160052729 |
| ACSM2B | 1.830655204 | 1.391997815 | 0.16392306 |
| RIMBP2 | 1.74589315 | 1.390600989 | 0.164346456 |
| TP53 | 1.812637312 | 1.382618637 | 0.166781831 |
| IGFBP2 | 1.908913044 | 1.379669508 | 0.167688425 |
| DOCK3 | 0.371975359 | -1.375879679 | 0.168858887 |
| AMPH | 0.442741585 | -1.374410218 | 0.169314366 |
| ANO1 | 1.733311539 | 1.372349667 | 0.169954611 |
| SLITRK2 | 0.446038615 | -1.363969855 | 0.172577053 |
| TINAG | 0.446509926 | -1.362007274 | 0.17319559 |
| HERC2 | 1.79473846 | 1.350366555 | 0.176898434 |
| ZP4 | 0.499292099 | -1.344919033 | 0.178651383 |
| SYNPO2 | 0.451989813 | -1.339239148 | 0.180492832 |
| MUC12 | 1.876439389 | 1.339001447 | 0.180570202 |
| STON1-GTF2A1L | 0.452413817 | -1.337711746 | 0.180990421 |
| IQSEC2 | 1.77340686 | 1.334211267 | 0.182134628 |
| PXDNL | 1.770449763 | 1.330570764 | 0.183330288 |
| CACNA1B | 1.701133173 | 1.325989176 | 0.184843283 |
| MUC16 | 1.385675434 | 1.317843544 | 0.187556028 |
| CCDC141 | 0.507247672 | -1.313403285 | 0.189047091 |
| NALCN | 1.602943599 | 1.309007311 | 0.190531874 |
| FLNC | 1.595359661 | 1.297662544 | 0.19440332 |
| RB1 | 1.474865142 | 1.289123647 | 0.197355104 |
| RLF | 1.675966153 | 1.286006939 | 0.19844064 |
| MME | 1.735985462 | 1.283675766 | 0.199255427 |
| FLG2 | 0.397562797 | -1.283447162 | 0.19933546 |
| ODZ2 | 1.745317797 | 1.280953265 | 0.200210082 |
| TMPRSS15 | 0.55217587 | -1.273971369 | 0.202673563 |
| RYR2 | 0.730067513 | -1.270848045 | 0.203782713 |
| DNAH7 | 0.619289309 | -1.269559892 | 0.204241444 |
| TACR3 | 0.47224437 | -1.268838238 | 0.204498764 |
| EPG5 | 0.518730094 | -1.265481154 | 0.2056989 |
| SCN11A | 0.470588003 | -1.265228226 | 0.205789527 |
| COL11A2 | 0.518891845 | -1.263065664 | 0.206565582 |
| IL1RAPL2 | 1.809286002 | 1.261317608 | 0.20719444 |
| PANX3 | 0.404396935 | -1.259996345 | 0.207670681 |
| PDE4DIP | 0.474621216 | -1.259482148 | 0.207856234 |
| DCHS1 | 0.556970353 | -1.2554046 | 0.20933192 |
| PCDHA3 | 0.476856486 | -1.251127315 | 0.210888031 |
| ANO4 | 1.796472141 | 1.250659197 | 0.211058843 |
| RBMXL3 | 1.789626406 | 1.24796384 | 0.2120443 |
| CNGB3 | 0.526337932 | -1.241857129 | 0.21428928 |
| SI | 1.411837811 | 1.224727769 | 0.220677808 |
| SCN1A | 1.557467036 | 1.223826468 | 0.221017697 |
| SVEP1 | 0.643637647 | -1.223694118 | 0.221067639 |
| INSRR | 0.485781412 | -1.221149866 | 0.222029282 |
| LYST | 0.533130778 | -1.217889233 | 0.223266068 |
| PTCHD2 | 1.769203667 | 1.215442976 | 0.224197185 |
| DUSP27 | 0.418681623 | -1.210867757 | 0.225946092 |
| CDH12 | 1.548942861 | 1.210070389 | 0.226251884 |
| POM121L2 | 1.624718828 | 1.202796296 | 0.229055159 |
| SPAG17 | 1.619233254 | 1.201764061 | 0.229454953 |
| PCDHA6 | 0.539278907 | -1.195667922 | 0.231826174 |
| F5 | 1.537612113 | 1.194966816 | 0.232099995 |
| P2RY10 | 1.857143003 | 1.193047483 | 0.232850778 |
| GRIA4 | 0.540061536 | -1.192476835 | 0.233074329 |
| ZFHX4 | 1.343630531 | 1.191921822 | 0.233291901 |
| NRCAM | 0.49522814 | -1.185952381 | 0.235641106 |
| MUC4 | 0.577552333 | -1.176180202 | 0.239522874 |
| ITIH6 | 1.559883198 | 1.175596255 | 0.239756253 |
| COL9A1 | 0.577951308 | -1.175402749 | 0.239833624 |
| OR4K5 | 0.49961327 | -1.171914497 | 0.241231386 |
| PDZRN4 | 1.552932519 | 1.163105719 | 0.244786612 |
| MYOM2 | 0.549024627 | -1.160386015 | 0.245891678 |
| FOXA2 | 1.722776241 | 1.159914721 | 0.246083528 |
| ACAN | 1.555217331 | 1.159006293 | 0.24645362 |
| LIPI | 0.501873851 | -1.155966783 | 0.247694744 |
| CNTNAP4 | 1.645309861 | 1.152877708 | 0.248960583 |
| SCN10A | 0.551651714 | -1.15265068 | 0.249053792 |
| SLFN11 | 1.708147077 | 1.13999988 | 0.254286351 |
| TRDN | 1.507165815 | 1.139672326 | 0.254422841 |
| MUC22 | 1.697639361 | 1.134193057 | 0.256713576 |
| HIVEP3 | 1.57464685 | 1.132487653 | 0.257429472 |
| POM121L12 | 1.796222507 | 1.127895248 | 0.259364161 |
| FAT3 | 1.394719551 | 1.122836755 | 0.261506832 |
| IL9R | 1.568094575 | 1.119252842 | 0.263032288 |
| XPNPEP2 | 0.44767193 | -1.117929273 | 0.263597201 |
| SPHKAP | 1.413870774 | 1.116323392 | 0.264283732 |
| GREB1 | 1.618007491 | 1.116027702 | 0.264410277 |
| ZNF804B | 1.427328043 | 1.112649867 | 0.265858835 |
| RIMS1 | 1.446158873 | 1.1113063 | 0.266436529 |
| UNC13C | 1.560750026 | 1.110759814 | 0.266671748 |
| OR2G2 | 0.564018606 | -1.106997369 | 0.268295066 |
| HECW2 | 1.55863531 | 1.106955955 | 0.268312972 |
| LRRC66 | 0.565889511 | -1.101517855 | 0.270671338 |
| EP400 | 1.460139229 | 1.098983306 | 0.271775348 |
| KIAA2022 | 1.667847349 | 1.097274872 | 0.272521252 |
| ZIM3 | 1.659793487 | 1.081643385 | 0.279411019 |
| TTC24 | 1.656044415 | 1.077193202 | 0.281393962 |
| STAB2 | 1.473958642 | 1.075727851 | 0.282048988 |
| IQSEC3 | 0.531025465 | -1.068976012 | 0.285080479 |
| FPGT-TNNI3K | 0.464704719 | -1.066151175 | 0.286355309 |
| ATP4A | 1.641827772 | 1.06358933 | 0.287514778 |
| LRRC7 | 1.420215196 | 1.058304347 | 0.289916712 |
| SLCO5A1 | 0.534687508 | -1.057956964 | 0.290075063 |
| KIF4B | 0.536945889 | -1.050370235 | 0.293547925 |
| HEPH | 0.583416399 | -1.04089666 | 0.297923512 |
| PCDHB11 | 0.540087938 | -1.040491557 | 0.298111585 |
| CACNA1H | 1.433809158 | 1.035944492 | 0.300228039 |
| SLC9A4 | 1.711230151 | 1.033265381 | 0.301479723 |
| MYO7B | 1.617816046 | 1.029997746 | 0.303011064 |
| UGT2B4 | 1.61519899 | 1.028682016 | 0.303629124 |
| SELP | 0.54347648 | -1.028614742 | 0.303660748 |
| REG3A | 0.543856313 | -1.028547738 | 0.303692247 |
| NEB | 1.712881313 | 1.027431495 | 0.304217328 |
| DNAH6 | 0.619791251 | -1.025698369 | 0.305033784 |
| LRRTM1 | 1.556926158 | 1.025665143 | 0.30504945 |
| SNAP91 | 0.544943196 | -1.02405648 | 0.305808599 |
| KIF1A | 0.545963863 | -1.022815065 | 0.306395294 |
| CRHBP | 1.69874443 | 1.019579055 | 0.307928142 |
| CTNND2 | 0.622389963 | -1.016234419 | 0.30951777 |
| ABCC11 | 0.591371519 | -1.015654119 | 0.309794124 |
| OR2M4 | 1.549033471 | 1.014259776 | 0.310458812 |
| ROBO3 | 0.621446917 | -1.014111979 | 0.310529323 |
| NOTCH3 | 1.512386296 | 1.013716973 | 0.310717823 |
| LRRIQ1 | 0.591638675 | -1.013557822 | 0.310793792 |
| TNRC18 | 0.593571021 | -1.010003838 | 0.312493451 |
| DPYD | 1.601850978 | 1.009521312 | 0.312724686 |
| ABCB5 | 1.602153594 | 1.006946986 | 0.313960251 |
| GRM5 | 0.626238611 | -1.003869813 | 0.315441369 |
| PCF11 | 0.552086073 | -1.002148111 | 0.316272065 |
| LAMB4 | 0.552619164 | -1.000009444 | 0.317305938 |
| C7orf58 | 1.461300897 | 0.996057707 | 0.319222108 |
| GNAS | 0.597865591 | -0.996050493 | 0.319225612 |
| ZFHX3 | 0.557816259 | -0.987091347 | 0.32359786 |
| FSIP2 | 1.335906537 | 0.981717907 | 0.326238841 |
| PARP8 | 0.4953555 | -0.977707995 | 0.328218766 |
| SCAND3 | 0.562296212 | -0.973477559 | 0.330316008 |
| ZNF479 | 1.444735575 | 0.972991924 | 0.330557316 |
| ODZ1 | 1.445135918 | 0.971739284 | 0.331180268 |
| C6orf103 | 0.725922383 | -0.968752932 | 0.332668476 |
| FBN3 | 0.637403357 | -0.967124149 | 0.333481974 |
| PLA1A | 1.514722899 | 0.965104542 | 0.334492452 |
| ADAMTS16 | 0.638430117 | -0.962774103 | 0.3356609 |
| COL4A1 | 0.639888231 | -0.959686788 | 0.337212875 |
| GPR158 | 0.5672977 | -0.958320462 | 0.337901189 |
| KLHL34 | 0.570044332 | -0.948833267 | 0.342705419 |
| PTPRQ | 0.636952874 | -0.947083359 | 0.343596304 |
| UBR4 | 1.460643965 | 0.946406457 | 0.343941313 |
| LRP1B | 0.791781614 | -0.942448076 | 0.345963283 |
| TG | 0.512493094 | -0.930351587 | 0.352189076 |
| HCN4 | 0.576698151 | -0.928371887 | 0.353214696 |
| CHRM2 | 1.540881167 | 0.927797245 | 0.353512753 |
| CACNA1E | 0.727594084 | -0.925454085 | 0.354729757 |
| RIF1 | 1.48425215 | 0.920295528 | 0.357418345 |
| FCGBP | 0.707137822 | -0.917805628 | 0.358720635 |
| MLL2 | 1.310811633 | 0.917133727 | 0.359072569 |
| MADD | 0.516865501 | -0.916896835 | 0.359196703 |
| MYO16 | 0.652404188 | -0.915888694 | 0.359725277 |
| WDR17 | 0.622989784 | -0.915496894 | 0.359930832 |
| INTS1 | 0.623042847 | -0.915181083 | 0.360096574 |
| CCDC168 | 1.354444209 | 0.915130719 | 0.36012301 |
| TNN | 1.355248265 | 0.913842169 | 0.360799785 |
| NOTCH1 | 0.720108614 | -0.91331581 | 0.361076469 |
| DNHD1 | 1.527761658 | 0.909066322 | 0.363315118 |
| FRYL | 0.585245872 | -0.905660319 | 0.365115672 |
| THOC2 | 0.656052369 | -0.903946993 | 0.366023509 |
| SORCS3 | 1.473288343 | 0.903532319 | 0.366243443 |
| NELL1 | 1.526043053 | 0.903361332 | 0.366334155 |
| ANK3 | 1.476169892 | 0.902694639 | 0.366687982 |
| PCNT | 1.589848227 | 0.894252962 | 0.371186561 |
| MYO7A | 0.630014878 | -0.894039151 | 0.371300944 |
| CD163L1 | 0.682942955 | -0.888279014 | 0.374390685 |
| CRB2 | 1.512334329 | 0.886976181 | 0.375091724 |
| MYBPC1 | 1.513753726 | 0.886471124 | 0.375363708 |
| OR2L8 | 1.516594081 | 0.886114009 | 0.375556095 |
| FGF23 | 1.582531026 | 0.885555539 | 0.375857079 |
| KIDINS220 | 0.633014714 | -0.885366889 | 0.375958785 |
| CHD3 | 0.592994627 | -0.88325261 | 0.377099802 |
| MYH14 | 0.593401977 | -0.882175199 | 0.377682072 |
| LILRB2 | 0.595770993 | -0.874738824 | 0.381716031 |
| AHNAK | 1.504990299 | 0.871004868 | 0.383751495 |
| SAMD1 | 1.366596125 | 0.868184178 | 0.385293514 |
| WNK3 | 0.599039306 | -0.86419349 | 0.387481608 |
| GOLGB1 | 0.641246353 | -0.860101255 | 0.38973323 |
| OR2T6 | 1.412873927 | 0.859491807 | 0.390069238 |
| ZFPM2 | 0.670312825 | -0.858320784 | 0.390715356 |
| C11orf41 | 1.490390284 | 0.855642839 | 0.392195367 |
| FAM135B | 1.282620019 | 0.855285099 | 0.392393335 |
| ASPM | 1.380904126 | 0.853283309 | 0.393502214 |
| PTPRH | 0.603579565 | -0.852379267 | 0.394003624 |
| MEGF8 | 0.644151174 | -0.851106171 | 0.394710378 |
| FCRL1 | 1.488997194 | 0.848933496 | 0.395918299 |
| ATP8A1 | 1.48705968 | 0.847666709 | 0.396623612 |
| CXorf22 | 1.486157874 | 0.846999742 | 0.396995265 |
| GPR174 | 0.67617611 | -0.839494574 | 0.401191833 |
| PHEX | 0.6087315 | -0.838888499 | 0.401531882 |
| PKHD1 | 1.286704352 | 0.835064768 | 0.403681243 |
| OR4Q3 | 0.650251159 | -0.833464403 | 0.404582866 |
| MAP3K5 | 1.430153174 | 0.831360077 | 0.405770245 |
| OR51S1 | 1.426700007 | 0.828629281 | 0.407314214 |
| MUC5B | 1.285999054 | 0.828514503 | 0.407379185 |
| ERBB4 | 1.392181789 | 0.82599207 | 0.40880859 |
| DCHS2 | 1.430011784 | 0.821172488 | 0.411548025 |
| LILRA1 | 1.468648189 | 0.81820261 | 0.413241505 |
| SLC8A3 | 1.52554005 | 0.815420354 | 0.414831738 |
| ZIC1 | 1.416418638 | 0.809981235 | 0.417950961 |
| TFAP2D | 1.323065735 | 0.809112199 | 0.418450612 |
| KCNN2 | 1.520771942 | 0.808775476 | 0.418644305 |
| SCN7A | 1.414063843 | 0.807310226 | 0.419487774 |
| SLC44A5 | 1.457201069 | 0.80276559 | 0.422110234 |
| SHANK1 | 1.334808777 | 0.80258705 | 0.422213456 |
| GABRA2 | 0.625140222 | -0.794518646 | 0.426893558 |
| NOS1 | 1.40427525 | 0.791168012 | 0.428845955 |
| PTPRT | 1.405304777 | 0.79044143 | 0.429270015 |
| OR4C13 | 1.505698722 | 0.789394659 | 0.429881376 |
| MYT1 | 1.50532888 | 0.787969046 | 0.430714812 |
| PLXNA3 | 0.665981609 | -0.787032097 | 0.431263077 |
| XDH | 1.444324307 | 0.78494443 | 0.432486151 |
| DNAH8 | 0.693793994 | -0.784868149 | 0.432530879 |
| PKHD1L1 | 0.778798538 | -0.783371759 | 0.433408835 |
| ZEB2 | 1.442200456 | 0.782304958 | 0.434035374 |
| FBN2 | 1.29309669 | 0.776098943 | 0.437690568 |
| MYO18B | 0.69692034 | -0.77470683 | 0.438512914 |
| NAV3 | 1.225934647 | 0.768729758 | 0.442053755 |
| DNAH14 | 1.39109646 | 0.768620132 | 0.44211885 |
| SLITRK3 | 0.673178419 | -0.766381491 | 0.443449342 |
| SMARCA2 | 1.57690498 | 0.765205882 | 0.444148958 |
| SHROOM4 | 0.673674406 | -0.763109959 | 0.445397821 |
| DZIP3 | 1.356846666 | 0.760244669 | 0.447108349 |
| COL4A5 | 0.639200117 | -0.755164522 | 0.450150273 |
| SYNE1 | 0.792976504 | -0.748864438 | 0.453938916 |
| CNTNAP5 | 1.293048346 | 0.745979497 | 0.4556798 |
| RP1L1 | 0.680787896 | -0.744508285 | 0.456569031 |
| NRK | 0.706678006 | -0.74367694 | 0.457071944 |
| PRDM9 | 1.259179937 | 0.743528626 | 0.457161697 |
| PCLO | 0.782754208 | -0.741847975 | 0.45817945 |
| MYO10 | 1.374956713 | 0.741445136 | 0.458423586 |
| C12orf12 | 0.645646854 | -0.739945275 | 0.459333201 |
| BBX | 0.645529836 | -0.739842541 | 0.459395543 |
| ABCA2 | 1.372379244 | 0.737945296 | 0.460547692 |
| RGSL1 | 0.745574079 | -0.733838298 | 0.463047296 |
| CACNA1I | 0.730327055 | -0.732140417 | 0.464082867 |
| RGS7 | 0.711244079 | -0.731855426 | 0.464256815 |
| ZNF729 | 1.262040787 | 0.726543624 | 0.467505575 |
| ZNF257 | 0.594686299 | -0.722193585 | 0.470175467 |
| ZNF804A | 1.282528316 | 0.718345348 | 0.472544376 |
| HCN1 | 0.797386163 | -0.708296544 | 0.478761123 |
| CADPS2 | 1.391120212 | 0.707902916 | 0.479005549 |
| CEP170 | 1.390911413 | 0.707128992 | 0.479486321 |
| DOT1L | 1.445658566 | 0.707056634 | 0.479531284 |
| ZNF521 | 0.719543482 | -0.706835709 | 0.479668581 |
| MYH8 | 0.719399113 | -0.706390771 | 0.479945158 |
| TNRC6B | 1.330201124 | 0.705716445 | 0.480364492 |
| PRDM13 | 1.388485005 | 0.704760046 | 0.480959577 |
| ZNF469 | 1.325548723 | 0.703614314 | 0.481672995 |
| RNF17 | 1.35075148 | 0.700250437 | 0.483770918 |
| ABCA6 | 0.661122384 | -0.69900349 | 0.48454985 |
| KIT | 1.386634064 | 0.698894994 | 0.484617656 |
| SAMD3 | 0.722728531 | -0.696361082 | 0.486202729 |
| DGKB | 0.661903636 | -0.695823016 | 0.486539674 |
| SMG1 | 1.43293867 | 0.694041087 | 0.487656443 |
| MYH7 | 0.725640164 | -0.688034255 | 0.49143121 |
| COL11A1 | 1.194168517 | 0.680849387 | 0.495966797 |
| MTUS2 | 1.313717485 | 0.680358159 | 0.496277707 |
| C18orf34 | 0.668539012 | -0.680261356 | 0.496338989 |
| OR2W3 | 1.37498904 | 0.679678117 | 0.496708295 |
| ZNF407 | 1.293301368 | 0.67943896 | 0.496859771 |
| MCTP1 | 0.728861226 | -0.678188628 | 0.497652102 |
| ZFC3H1 | 0.705159088 | -0.675469664 | 0.499377418 |
| MYO3A | 0.763256546 | -0.674009221 | 0.500305451 |
| COL12A1 | 1.272370715 | 0.670051681 | 0.502824846 |
| KCNK10 | 0.709420889 | -0.664733709 | 0.506220829 |
| ZNF98 | 1.410401046 | 0.663267118 | 0.507159492 |
| PEAR1 | 1.409031305 | 0.662105239 | 0.507903778 |
| CSMD3 | 1.18434234 | 0.660900555 | 0.50867609 |
| CACNA1C | 1.326217379 | 0.656225078 | 0.511679316 |
| TGM3 | 1.401296133 | 0.652058198 | 0.514363629 |
| PTEN | 0.681676969 | -0.647839192 | 0.517088964 |
| DSP | 1.35177359 | 0.646129145 | 0.518195722 |
| SBSN | 0.682865622 | -0.64460307 | 0.519184445 |
| KIAA1324L | 0.683692044 | -0.64257837 | 0.520497722 |
| ZNF423 | 1.316534565 | 0.640903975 | 0.521585073 |
| ANK2 | 0.774675399 | -0.638216015 | 0.523333074 |
| OR8B4 | 1.387485251 | 0.631974534 | 0.527403518 |
| LPHN3 | 1.234493919 | 0.631556818 | 0.527676511 |
| MUT | 0.688910271 | -0.629261171 | 0.529178088 |
| ELTD1 | 1.341937761 | 0.629058022 | 0.529311071 |
| SCN2A | 1.31037246 | 0.628898093 | 0.529415774 |
| SCN3A | 1.306653581 | 0.623248707 | 0.533121099 |
| OTOGL | 0.808425463 | -0.619390835 | 0.535658917 |
| PTPRC | 0.693450109 | -0.6186316 | 0.536159079 |
| PEG3 | 0.780744075 | -0.618166191 | 0.536465794 |
| ZNF716 | 1.303333831 | 0.617173451 | 0.537120325 |
| DCAF8L2 | 1.261709351 | 0.614908856 | 0.538614914 |
| GABRB1 | 1.279078484 | 0.613457277 | 0.539574024 |
| ABCC8 | 0.728621334 | -0.612698082 | 0.540075991 |
| ATP10A | 0.729618675 | -0.609384108 | 0.542269869 |
| CENPE | 1.37092893 | 0.608233275 | 0.543032769 |
| LRRK2 | 1.233223384 | 0.605571291 | 0.544799471 |
| DOCK2 | 0.731041224 | -0.605298931 | 0.544980391 |
| GBA3 | 0.698876821 | -0.604758754 | 0.545339303 |
| NLRP4 | 1.295255601 | 0.603171709 | 0.546394466 |
| GCN1L1 | 0.754565739 | -0.603003415 | 0.546506417 |
| FSCB | 1.324320342 | 0.600191446 | 0.548378654 |
| DMD | 0.819846233 | -0.599929543 | 0.548553193 |
| TOPBP1 | 0.7328951 | -0.598586093 | 0.549448932 |
| HECW1 | 1.240339756 | 0.596038135 | 0.551149751 |
| KCNT2 | 1.238396798 | 0.59320743 | 0.553042341 |
| GAD2 | 1.317135331 | 0.590818295 | 0.554642176 |
| RTN1 | 0.737511353 | -0.589173188 | 0.555745101 |
| DLGAP2 | 1.315881302 | 0.588258626 | 0.556358712 |
| MRGPRX4 | 1.315949499 | 0.587246277 | 0.557038316 |
| PLXNB3 | 1.31111668 | 0.581524638 | 0.560886918 |
| KIF2B | 0.779889819 | -0.578991889 | 0.562594646 |
| KCNQ3 | 1.347471936 | 0.577009174 | 0.563933258 |
| HIVEP2 | 0.76522012 | -0.573988282 | 0.565975731 |
| THSD7A | 1.2784571 | 0.571960764 | 0.567348556 |
| MDN1 | 1.340214878 | 0.566542299 | 0.571025187 |
| DSCAML1 | 0.746350965 | -0.565845609 | 0.571498739 |
| ITGAL | 1.300583329 | 0.5631904 | 0.573305239 |
| ABCA13 | 1.213978333 | 0.563169399 | 0.573319539 |
| BSN | 1.299921616 | 0.562154355 | 0.574010857 |
| TMEM132E | 1.299080815 | 0.561076981 | 0.57474506 |
| VPS13C | 0.718754704 | -0.558196932 | 0.576709916 |
| MUC17 | 1.184693452 | 0.557852593 | 0.576945046 |
| ANKRD30A | 1.232527555 | 0.553183398 | 0.580137834 |
| FN1 | 0.751639941 | -0.552744828 | 0.580438153 |
| LRP1 | 0.721682034 | -0.551333605 | 0.581405006 |
| VPS13A | 0.752620999 | -0.550021428 | 0.582304676 |
| CNTNAP2 | 0.753029599 | -0.549372169 | 0.58275007 |
| CDH9 | 1.198539592 | 0.546045581 | 0.585034605 |
| C1orf173 | 1.174265105 | 0.545955807 | 0.585096315 |
| DCAF4L2 | 1.326237397 | 0.54501999 | 0.585739769 |
| SLC39A12 | 1.326255142 | 0.54427421 | 0.586252792 |
| CEP350 | 0.724761339 | -0.543953756 | 0.586473296 |
| PTPRM | 1.323035211 | 0.539741688 | 0.589375186 |
| ACTN2 | 1.284444119 | 0.536982484 | 0.591279713 |
| NRG3 | 0.728500316 | -0.535327991 | 0.592423074 |
| SBF1 | 0.758853573 | -0.534343774 | 0.593103712 |
| MYPN | 0.780347106 | -0.532429141 | 0.594428806 |
| LRFN5 | 0.82516113 | -0.53221522 | 0.594576943 |
| PCDHGA1 | 1.316594719 | 0.532132499 | 0.59463423 |
| F13A1 | 1.253769012 | 0.527152548 | 0.598087658 |
| KCNU1 | 0.78303737 | -0.524455913 | 0.599961476 |
| PTPRZ1 | 0.734420231 | -0.520757918 | 0.602535422 |
| HRNR | 0.784558098 | -0.520619286 | 0.602632012 |
| ZNF208 | 1.217425109 | 0.517918995 | 0.604514789 |
| DPP10 | 0.785797343 | -0.517588144 | 0.604745655 |
| CD1E | 0.73709329 | -0.515632177 | 0.606111333 |
| BAI3 | 1.204123448 | 0.513782774 | 0.607403873 |
| FAT2 | 0.740607809 | -0.507451529 | 0.611838038 |
| EPHA6 | 0.805646778 | -0.504531436 | 0.613887977 |
| DMXL1 | 0.742265505 | -0.503632047 | 0.614519968 |
| ASTN1 | 0.771256582 | -0.503142119 | 0.614864356 |
| ODZ3 | 0.805721467 | -0.503053369 | 0.614926751 |
| SPTB | 0.743080113 | -0.501239303 | 0.616202717 |
| ZAN | 1.295380663 | 0.500257068 | 0.61689408 |
| FLRT2 | 0.772228338 | -0.499277946 | 0.617583589 |
| GPR98 | 1.187239124 | 0.498442199 | 0.6181724 |
| CCDC108 | 0.792759571 | -0.498266952 | 0.618295898 |
| NLRP8 | 0.807301309 | -0.498182785 | 0.618355215 |
| GTF3C1 | 0.808150616 | -0.497089301 | 0.619126079 |
| NRXN1 | 0.830300851 | -0.49209211 | 0.62265423 |
| ADAMTS20 | 0.821135499 | -0.492012357 | 0.622710608 |
| LAMA5 | 0.821841405 | -0.489606477 | 0.624412392 |
| CXorf59 | 0.777341838 | -0.487191701 | 0.626122486 |
| FER1L6 | 0.749986017 | -0.486288664 | 0.626762515 |
| TRIO | 1.254210012 | 0.486255541 | 0.626785996 |
| CACNA1A | 0.824253559 | -0.48259614 | 0.62938252 |
| C5orf42 | 0.813745855 | -0.479477252 | 0.631599148 |
| UNC5D | 1.227482808 | 0.477166149 | 0.633243814 |
| COL5A2 | 0.754014039 | -0.477134116 | 0.633266623 |
| HS3ST4 | 0.800519842 | -0.477030325 | 0.633340528 |
| FCRL5 | 1.226949347 | 0.476145479 | 0.633970739 |
| OR2G6 | 1.225483369 | 0.474216178 | 0.635345758 |
| TYR | 0.782583276 | -0.474166966 | 0.635380848 |
| FAM5B | 1.277862301 | 0.473394429 | 0.635931804 |
| EPHA5 | 0.829023034 | -0.468331144 | 0.6395478 |
| NPY1R | 0.757966727 | -0.468262471 | 0.639596903 |
| KIAA1210 | 1.242838045 | 0.466487918 | 0.640866294 |
| POTEF | 0.805772388 | -0.463331997 | 0.643126414 |
| ALPK3 | 0.787603197 | -0.462016195 | 0.644069707 |
| FPGT | 0.787722479 | -0.461612896 | 0.644358945 |
| COL4A3 | 1.21921 | 0.461560902 | 0.644396238 |
| ALK | 0.787971467 | -0.460815412 | 0.644931044 |
| PRUNE2 | 1.239249943 | 0.459514621 | 0.645864655 |
| GRIA3 | 1.238673019 | 0.457217065 | 0.647515034 |
| DOCK10 | 0.822152906 | -0.456353865 | 0.648135535 |
| ZNF135 | 0.764081725 | -0.454306769 | 0.649608045 |
| VWA5B1 | 1.265119121 | 0.453646087 | 0.650083577 |
| DNAH9 | 1.198472722 | 0.451519291 | 0.651615325 |
| DSCAM | 0.793481066 | -0.447665743 | 0.654394448 |
| ITPR2 | 0.81184653 | -0.447043958 | 0.654843322 |
| CD1C | 0.793872449 | -0.446520905 | 0.655221015 |
| DSG4 | 0.79439666 | -0.445718866 | 0.655800335 |
| HEATR7B2 | 1.157701707 | 0.441318998 | 0.658982074 |
| LEPR | 0.79650663 | -0.440100844 | 0.65986407 |
| GABRG1 | 0.828250133 | -0.439421572 | 0.660356098 |
| DCC | 1.224617413 | 0.434472364 | 0.663945459 |
| NLRP14 | 0.848860777 | -0.432764816 | 0.665185637 |
| CEP290 | 1.223174796 | 0.431140003 | 0.666366575 |
| MMP24 | 0.775583815 | -0.429482702 | 0.66757198 |
| TTC28 | 0.801978384 | -0.426664317 | 0.669623844 |
| FMN2 | 0.872329737 | -0.426650829 | 0.66963367 |
| OR2C3 | 1.219658117 | 0.425468034 | 0.67049552 |
| IGSF1 | 1.173940416 | 0.424508662 | 0.67119489 |
| SLC14A2 | 0.803901645 | -0.421841706 | 0.673140561 |
| RUNX1T1 | 0.804351005 | -0.421636729 | 0.673290193 |
| PLCB1 | 0.779943772 | -0.42007448 | 0.674431045 |
| ATRX | 0.80636704 | -0.416583883 | 0.6769828 |
| RYR3 | 1.133578244 | 0.415517005 | 0.677763469 |
| HUWE1 | 0.846661825 | -0.415366171 | 0.677873867 |
| PCDH17 | 1.159927644 | 0.41335123 | 0.679349301 |
| ODZ4 | 0.825387524 | -0.411652395 | 0.680594222 |
| COL20A1 | 0.827734669 | -0.405451862 | 0.685145396 |
| TKTL2 | 0.840775272 | -0.404592824 | 0.685776834 |
| TDRD5 | 0.81473442 | -0.396641563 | 0.691631799 |
| DPP6 | 1.160257816 | 0.392067519 | 0.695008327 |
| UMOD | 0.794025278 | -0.389577352 | 0.696849102 |
| FAT4 | 0.846740324 | -0.387754077 | 0.698198033 |
| MGAM | 0.846806696 | -0.387506778 | 0.698381068 |
| FREM2 | 1.197486745 | 0.386015059 | 0.699485516 |
| FREM1 | 0.81989397 | -0.383530415 | 0.701326523 |
| GRM8 | 1.178609061 | 0.381541728 | 0.702801314 |
| HERC1 | 0.821652357 | -0.380283829 | 0.703734738 |
| FAM5C | 0.872902202 | -0.378289363 | 0.705215646 |
| OGDHL | 0.799173905 | -0.378160467 | 0.705311391 |
| PIK3C2G | 0.823888842 | -0.373121741 | 0.709057838 |
| KCNQ2 | 1.207037572 | 0.363978229 | 0.715874267 |
| TNR | 1.12338736 | 0.363458342 | 0.716262527 |
| PCDHB8 | 0.806261745 | -0.363426775 | 0.716286103 |
| COL19A1 | 0.855322779 | -0.363382602 | 0.716319096 |
| COLEC12 | 1.184629614 | 0.363125529 | 0.716511115 |
| TSHZ3 | 1.184815311 | 0.363096613 | 0.716532714 |
| CA2 | 0.829857247 | -0.361218529 | 0.717936092 |
| SPEF2 | 0.878811868 | -0.359831638 | 0.718973042 |
| EPHA7 | 1.182386055 | 0.359205739 | 0.719441184 |
| TCHH | 1.181656893 | 0.357557866 | 0.720674215 |
| CUBN | 0.867409918 | -0.355369092 | 0.722313101 |
| CNKSR2 | 1.164000755 | 0.354330877 | 0.723090931 |
| OR11L1 | 1.161363426 | 0.348712438 | 0.727305205 |
| NPAS3 | 1.175566521 | 0.346615784 | 0.728879986 |
| LRP2 | 1.160196633 | 0.346341459 | 0.729086114 |
| MYH13 | 0.870527461 | -0.346053693 | 0.729302364 |
| ANO5 | 1.174838179 | 0.345283647 | 0.72988114 |
| ROS1 | 0.851933145 | -0.343162792 | 0.731475995 |
| COBL | 1.173448022 | 0.34301787 | 0.731585016 |
| NFATC2 | 1.171138582 | 0.339040397 | 0.734579298 |
| TMEM200A | 0.85380222 | -0.338910082 | 0.734677469 |
| UNC79 | 0.880187715 | -0.337767463 | 0.735538431 |
| C15orf2 | 1.142626119 | 0.332381518 | 0.739601192 |
| CLASP1 | 0.823739865 | -0.327034704 | 0.743641637 |
| MLL4 | 0.869835129 | -0.325572343 | 0.74474794 |
| GRM7 | 1.16367905 | 0.32536738 | 0.74490304 |
| OR6F1 | 0.825407386 | -0.323712783 | 0.746155492 |
| PRKCB | 1.180330828 | 0.320581583 | 0.748527496 |
| FRAS1 | 1.160630254 | 0.319719891 | 0.749180679 |
| GPR112 | 0.872453552 | -0.318120055 | 0.750393872 |
| DACH2 | 1.158873248 | 0.316522105 | 0.75160625 |
| SPTA1 | 1.11356847 | 0.312810888 | 0.754424343 |
| CHD7 | 0.86452904 | -0.312002765 | 0.755038422 |
| ANK1 | 1.203769587 | 0.311957981 | 0.755072457 |
| SPEG | 0.852041732 | -0.309647604 | 0.756828951 |
| APOB | 0.884682253 | -0.30586302 | 0.759708943 |
| ARHGEF10 | 1.138715828 | 0.302489381 | 0.762279027 |
| MS4A14 | 0.868605573 | -0.302254155 | 0.762458324 |
| TRPM2 | 1.151020112 | 0.300832025 | 0.763542586 |
| PCDHB6 | 1.167281034 | 0.298808575 | 0.765086108 |
| FAT1 | 0.89325569 | -0.298288283 | 0.765483147 |
| HTR1A | 0.857656979 | -0.296684243 | 0.766707588 |
| MORC1 | 1.135958451 | 0.296306466 | 0.766996048 |
| CDH19 | 0.858911891 | -0.294434342 | 0.768426028 |
| MUC6 | 0.881603643 | -0.293954281 | 0.768792839 |
| FNDC1 | 1.105898072 | 0.293119804 | 0.76943058 |
| BRWD3 | 1.163639388 | 0.292684923 | 0.769762995 |
| ABCB1 | 0.840977606 | -0.2925425 | 0.76987187 |
| KIF26A | 1.145683113 | 0.291447749 | 0.770708901 |
| PCDHA8 | 1.162589988 | 0.291126221 | 0.770954787 |
| TPTE | 0.900825236 | -0.290451273 | 0.771471023 |
| GRM1 | 0.897442148 | -0.286566971 | 0.774443907 |
| WDFY4 | 0.885028203 | -0.284206987 | 0.776251761 |
| DYNC2H1 | 1.128777016 | 0.282241555 | 0.777758296 |
| EP300 | 0.900581469 | -0.275810802 | 0.782693387 |
| FAAH2 | 0.851436348 | -0.27170202 | 0.785851148 |
| PTPRB | 0.88989403 | -0.271244911 | 0.786202674 |
| SDK2 | 0.88160612 | -0.27055323 | 0.786734672 |
| TMEM132D | 0.923732538 | -0.270185174 | 0.787017798 |
| ATP1A2 | 1.148224497 | 0.266652285 | 0.789736895 |
| COL6A5 | 1.100849545 | 0.266490528 | 0.789861454 |
| DLG2 | 0.871792004 | -0.265548261 | 0.790587137 |
| ITGA7 | 0.883569026 | -0.265457488 | 0.790657055 |
| AKAP9 | 0.856051377 | -0.262586962 | 0.79286895 |
| COL22A1 | 0.918168347 | -0.256950199 | 0.797217218 |
| EPHB1 | 0.887856247 | -0.255313697 | 0.798480822 |
| DYSF | 0.888197069 | -0.253758957 | 0.799681783 |
| MKI67 | 0.87774142 | -0.252184598 | 0.800898382 |
| KIAA1239 | 0.889890007 | -0.250475248 | 0.802219844 |
| PCDHA13 | 1.123502968 | 0.24980455 | 0.802738501 |
| LAMA2 | 0.910234238 | -0.249368067 | 0.803076084 |
| DSPP | 1.102076161 | 0.241846994 | 0.808898724 |
| TMC2 | 1.133579849 | 0.241767809 | 0.808960084 |
| ADCY10 | 1.115545319 | 0.23463284 | 0.814493718 |
| DNER | 1.128248352 | 0.233340166 | 0.815497271 |
| ADAD1 | 0.886960516 | -0.232331253 | 0.81628074 |
| PCDH10 | 1.114133045 | 0.231285342 | 0.817093134 |
| AFF2 | 1.084939312 | 0.226304359 | 0.820964708 |
| BTBD11 | 1.101437618 | 0.224729271 | 0.822189892 |
| KLHL4 | 0.900697916 | -0.223374697 | 0.823243896 |
| KANK4 | 0.891178363 | -0.223047002 | 0.823498926 |
| OR4C46 | 1.100389299 | 0.222910202 | 0.823605396 |
| BPTF | 0.90319171 | -0.218619179 | 0.826946712 |
| F8 | 1.085430864 | 0.216880305 | 0.828301627 |
| MUC2 | 1.080506773 | 0.215252557 | 0.829570417 |
| KIF16B | 1.117077735 | 0.214390591 | 0.830242479 |
| FREM3 | 1.089396707 | 0.213718617 | 0.830766492 |
| F13B | 0.89645578 | -0.21146172 | 0.832527 |
| CEP192 | 1.103493034 | 0.210843316 | 0.833009537 |
| SLIT2 | 0.90723482 | -0.208524282 | 0.834819625 |
| AQPEP | 0.908295613 | -0.206445319 | 0.836443073 |
| TCN1 | 1.111169131 | 0.203678727 | 0.838604567 |
| RYR1 | 0.941501899 | -0.200190419 | 0.84133166 |
| C9orf79 | 1.089530375 | 0.199800554 | 0.841636568 |
| KCNT1 | 1.088227645 | 0.197180243 | 0.843686491 |
| PLEC | 0.919057664 | -0.196906137 | 0.843900991 |
| ASTN2 | 0.913806835 | -0.193434269 | 0.846618879 |
| TRIM58 | 1.084521002 | 0.189251127 | 0.849895998 |
| KCNH7 | 1.069992703 | 0.188484898 | 0.850496553 |
| MYLK | 1.07765317 | 0.18653784 | 0.852023008 |
| DNAH5 | 0.942319668 | -0.186265591 | 0.85223649 |
| TEP1 | 1.070442352 | 0.179843403 | 0.857275509 |
| WBSCR17 | 0.925901825 | -0.179352357 | 0.857661038 |
| KIAA1211 | 1.060998678 | 0.178989093 | 0.857946266 |
| CHAT | 1.071801537 | 0.173110626 | 0.862564473 |
| PCNX | 0.916725703 | -0.168212194 | 0.866416348 |
| C7 | 0.926377971 | -0.164182237 | 0.869587684 |
| CASR | 0.919593267 | -0.162007457 | 0.871299981 |
| ALMS1 | 0.937414656 | -0.161197661 | 0.871937723 |
| TRPM8 | 1.078007423 | 0.161194112 | 0.871940519 |
| OR2T2 | 0.927701799 | -0.160963652 | 0.872122029 |
| KCNH1 | 0.920308438 | -0.160650582 | 0.872368614 |
| LILRB5 | 0.93386069 | -0.159460894 | 0.873305766 |
| UFL1 | 1.076533245 | 0.158090037 | 0.874385851 |
| ZEB1 | 0.939218 | -0.156288442 | 0.875805666 |
| CPS1 | 0.922591934 | -0.155666046 | 0.876296262 |
| XIRP2 | 0.957415564 | -0.150856083 | 0.880089244 |
| ADAMTSL1 | 1.080177263 | 0.149267904 | 0.88134224 |
| OR2T1 | 1.057219845 | 0.147469621 | 0.882761357 |
| SGCZ | 0.928911748 | -0.142680228 | 0.886542733 |
| FAM47C | 0.944442768 | -0.142549666 | 0.886645852 |
| OR5J2 | 1.075507835 | 0.140799535 | 0.888028311 |
| HDAC9 | 1.075058784 | 0.139898192 | 0.888740429 |
| USH2A | 0.965976903 | -0.137785068 | 0.890410286 |
| PTPN13 | 1.072619619 | 0.13575486 | 0.892015077 |
| RASGRF2 | 1.072117651 | 0.134520395 | 0.892991085 |
| PRRC2C | 0.93310073 | -0.13404689 | 0.893365496 |
| CUX2 | 1.081952551 | 0.132804226 | 0.894348212 |
| NCAM2 | 0.959457764 | -0.129807558 | 0.896718683 |
| XRN1 | 0.94694035 | -0.1270974 | 0.898863309 |
| CDH10 | 1.044272206 | 0.126190681 | 0.899580988 |
| PAPPA | 1.067669033 | 0.12609168 | 0.899659353 |
| CMYA5 | 0.947445985 | -0.125833071 | 0.899864063 |
| MSH4 | 0.938353316 | -0.123099734 | 0.902028122 |
| PREX2 | 1.048585958 | 0.1184762 | 0.905690352 |
| NDST4 | 1.063009587 | 0.118222821 | 0.905891108 |
| HEPHL1 | 0.941666493 | -0.116158795 | 0.907526693 |
| CDH18 | 0.957222586 | -0.109182238 | 0.913057948 |
| GRIN2A | 1.047810396 | 0.108797938 | 0.913362759 |
| ZNF831 | 0.963372838 | -0.103715177 | 0.917395382 |
| LCT | 1.039611097 | 0.102960823 | 0.917994064 |
| COL5A3 | 1.046950591 | 0.098512938 | 0.921524999 |
| ATP6V0D2 | 1.051615464 | 0.097391459 | 0.922415527 |
| CCDC39 | 0.951649123 | -0.095851863 | 0.923638226 |
| EGF | 1.050614312 | 0.095562266 | 0.923868235 |
| COL4A2 | 0.957713907 | -0.083565739 | 0.933401708 |
| SYNE2 | 1.03033442 | 0.08314349 | 0.933737445 |
| NLRP11 | 1.042915867 | 0.081280583 | 0.935218816 |
| CDH7 | 0.960101491 | -0.078667372 | 0.937297199 |
| PTPRD | 1.028516199 | 0.078323332 | 0.937570858 |
| NELL2 | 0.962364872 | -0.074213475 | 0.940840524 |
| ADCY8 | 0.974519953 | -0.071958623 | 0.942634836 |
| D2HGDH | 1.03384934 | 0.071313868 | 0.943147958 |
| OR5M9 | 1.035557097 | 0.067542808 | 0.946149584 |
| ANO3 | 1.031072044 | 0.065595744 | 0.947699677 |
| TRPM6 | 0.970416383 | -0.064496465 | 0.948574921 |
| FSCN3 | 1.027633224 | 0.063586685 | 0.949299334 |
| ZNF99 | 0.970858632 | -0.063183287 | 0.949620553 |
| EYS | 1.018335844 | 0.061936978 | 0.95061302 |
| FLG | 1.021111382 | 0.060865363 | 0.951466435 |
| PLEKHH2 | 0.97278606 | -0.059080694 | 0.952887836 |
| OR8K1 | 0.970029639 | -0.058680107 | 0.953206904 |
| FLNA | 0.973007151 | -0.058643411 | 0.953236133 |
| CNTN5 | 1.027404428 | 0.057678234 | 0.954004932 |
| TMEM132C | 1.018762273 | 0.056203911 | 0.955179366 |
| OR5L2 | 0.971237306 | -0.056189317 | 0.955190992 |
| COL15A1 | 0.978679611 | -0.05365003 | 0.957213996 |
| TRIM63 | 0.97492399 | -0.049139143 | 0.96080841 |
| MAGEB6 | 1.025483217 | 0.048693406 | 0.961163631 |
| FAM123B | 0.975937678 | -0.047097082 | 0.962435853 |
| KCNC2 | 1.022183085 | 0.046839418 | 0.962641213 |
| SYN1 | 1.017877223 | 0.038038566 | 0.969656933 |
| KLHL1 | 1.017539078 | 0.037311431 | 0.970236692 |
| KRTAP10-11 | 1.017335448 | 0.036836484 | 0.970615384 |
| TRPS1 | 1.017207658 | 0.03658105 | 0.970819053 |
| FAM47A | 1.016993677 | 0.036187718 | 0.971132679 |
| MYT1L | 1.015852446 | 0.033702221 | 0.973114608 |
| PIK3CG | 1.015259427 | 0.032491403 | 0.974080172 |
| RELN | 0.987745951 | -0.032464611 | 0.974101538 |
| ADAMTS12 | 0.989655803 | -0.031378536 | 0.974967659 |
| MDGA2 | 0.98438955 | -0.030434726 | 0.97572035 |
| TRIM60 | 1.013664908 | 0.029123136 | 0.976766384 |
| TMEM132B | 0.985644296 | -0.027999283 | 0.977662723 |
| ZCCHC2 | 1.014396599 | 0.027470335 | 0.9780846 |
| MYH1 | 0.98927095 | -0.025165892 | 0.979922643 |
| OR5D14 | 1.011626467 | 0.022378106 | 0.982146345 |
| PCDH15 | 1.006044784 | 0.022169841 | 0.982312475 |
| PAPPA2 | 1.005528796 | 0.016673007 | 0.986697481 |
| PRKDC | 0.9925493 | -0.014462542 | 0.988460964 |
| OR5D13 | 1.006366675 | 0.013616639 | 0.98913583 |
| AKR1D1 | 0.995190832 | -0.010333522 | 0.991755189 |
| NLRP3 | 1.003659528 | 0.008483886 | 0.993230919 |
| GRID2 | 1.002677753 | 0.005737484 | 0.995422175 |
| GLT25D2 | 0.99850831 | -0.003192784 | 0.997452532 |
| SEMA5B | 0.998934574 | -0.002482188 | 0.998019503 |
| HTT | 1.001231027 | 0.002375028 | 0.998105003 |
| OR5L1 | 0.999058306 | -0.001823135 | 0.99854535 |
| EDIL3 | 0.999282488 | -0.001385069 | 0.998894875 |
| ANKS1B | 4.995447568 | 4.10036119 | 4.13E-05 |
| NDST3 | 9.059317222 | 4.523208341 | 6.09E-06 |
